# Antimicrobial-resistant bacteria in food: which behaviour change strategies increase consumers’ risk awareness and preventive food-handling behaviour?

**DOI:** 10.1080/21642850.2021.1912609

**Published:** 2021-04-20

**Authors:** Claudia Freivogel, Vivianne H. M. Visschers

**Affiliations:** School of Applied Psychology, University of Applied Sciences and Arts Northwestern Switzerland, Olten, Switzerland

**Keywords:** food-handling behaviour, antimicrobial-resistant bacteria, educational video, personalised risk message, goal setting

## Abstract

**Objectives:**

We aimed to identify the potential of behaviour change strategies to effectively increase consumers’ risk awareness, behavioural intention, and preventive food-handling behaviour to reduce the transmission risk of antimicrobial-resistant bacteria through food. The applied strategies targeted knowledge and determinants of the health action process approach (HAPA). We tested techniques that are expected to increase knowledge, risk perception, and positive outcome expectancy (Study 1) as well as those that increase planning and coping self-efficacy (Study 2) in two randomised control trials.

**Methods:**

In Study 1 (*N* = 328), a 2 × 2 between-subject design was employed to investigate the effects of an educational video about the spread of antimicrobial-resistant bacteria and a personalised risk message on knowledge, risk perception, outcome expectancy and consequently on intention regarding safe food-handling behaviour. In Study 2 (*N* = 129), we used a 2 × 2 design to examine to what extent goal setting (implemented vs. not implemented) and time (pre- vs. post-test) affected planning, coping self-efficacy, and preventive food-handling behaviour.

**Results:**

In Study 1, we found that the video increased knowledge and the perceived susceptibility of risk compared to the control video. We found no increase on the dependent variables after receiving the personalised risk message. In Study 2, goal setting significantly improved safe food-handling behaviour compared to the control condition. Moreover, participants in the goal-setting condition showed more planning of safe food-handling measures and of dealing with emerging barriers than participants in the control condition.

**Conclusions:**

These findings demonstrate that the delivery of an educational video on the spread of antimicrobial-resistant bacteria is a useful strategy to increase risk awareness, whereas goal setting presents a promising approach to improve food-handling behaviour. Following the HAPA, an additional effective behaviour change technique is required that decreases negative outcome expectancies and improves coping self-efficacy, thereby further improving intention and behaviour.

## Introduction

The presence of multi-drug antimicrobial-resistant pathogens in food of animal origin—such as meat, poultry, and milk—has dramatically increased over the last few years (Pérez-Rodríguez & Mercanoglu Taban, 2019). Infections caused by antimicrobial-resistant pathogens pose a particular risk to consumers because they are difficult to treat with or are even untreatable by antimicrobials. Samples from raw meat, milk, seafood, and certain fermented dairy products available on the Swiss retail market have featured the occurrence of various antimicrobial-resistant bacteria (Jans et al., 2018). Various foodborne outbreaks with multidrug-resistant bacteria (e.g. salmonellosis) have shown that antimicrobial-resistant bacteria transfer from poultry and meat to humans (e.g. Alt et al., 2015; Folster et al., 2017; Liu et al., 2016; Oakeson, Wagner, Rohrwasser, & Atkinson-Dunn, 2018; Xiang et al., 2020). Relevant exposure pathways are preparation and consumption of affected foods that were not properly handled (e.g. insufficiently heated) (WHO, 2008). Therefore, the spread of antimicrobial-resistant bacteria through food can be mitigated by adhering to certain hygiene measures. Hygiene measures, such as using different cutting boards for raw meat and other ingredients, significantly reduce the contamination level of foodborne pathogens (De Jong, Verhoeff-Bakkenes, Nauta, & De Jonge, 2008; Røssvoll et al., 2015), regardless of whether the latter are resistant or non-resistant against antimicrobials. More consumers need to be mindful of these measures when preparing food at home (Swiss Federal Food Safety and Veterinary Office, 2019). We therefore investigated the effectiveness of theory-based intervention strategies to improve awareness for antimicrobial resistance and to foster safe food-handling behaviour among consumers.

### Factors influencing behaviour change

According to stage models of behaviour change, individuals’ behaviour evolves over time (Prochaska & DiClemente, 1982; Schwarzer, 2008; Sniehotta & Aunger, 2010). Behaviour change usually requires movement through a sequence of discrete stages or phases, whereby different factors are assumed to be influential at different stages. Hence, people in different stages are assumed to require different types of interventions to encourage or help them to move to the next stage towards behaviour change. Here, we focus on the health action process approach (HAPA; Schwarzer, 1992), a dominant model in the field of health psychology, which has also been applied in predicting hygienic food handling to reduce the transmission risk of antimicrobial-resistant bacteria (Freivogel & Visschers, 2020). The HAPA describes a sequence of two self-regulatory processes that guide behaviour change: a pre-intentional motivational phase and a post-intentional volitional phase (Schwarzer, 1992). The latter can be further subdivided into a pre-action phase and an action phase.

In the motivational phase, the HAPA suggests three factors that predict the intention to change behaviour. The first factor is *risk perception,* which is defined as the perceived susceptibility to and the perceived severity of a health threat. Susceptibility refers to an individual’s perception of the likelihood of being infected with antimicrobial-resistant bacteria through food, whereas severity refers to an individual’s perception of the seriousness of the health consequences of an antimicrobial-resistant infection. The second pre-motivational factor is called *outcome expectancy*. It concerns a person’s beliefs regarding positive or negative consequences due to a certain behaviour. The final factor is *self-efficacy*, which can be defined as an individual’s belief in his/her own capacity to execute hygienic food-handling measures. High self-efficacy is required throughout the entire process, whereas the nature of self-efficacy differs from phase to phase (Schwarzer, 2016). Although knowledge is not an explicit factor in the HAPA, other models suggest that individuals with a low level of knowledge or problem awareness are assumed to be without an intention to change their behaviour (Prochaska & DiClemente, 1982).

Merely having an intention does not always result in behaviour change among people. This so-called intention-behaviour gap is well documented in the literature (e.g. Hassan, Shiu, & Shaw, 2016; Mullan, Wong, & Kothe, 2013; Rhodes & Dickau, 2012). Therefore, the HAPA suggests that people are required to enter the volitional phase in which *planning* serves as a mediator between intention and behaviour (Schwarzer, 2008; Wiedemann, Lippke, Reuter, Ziegelmann, & Schwarzer, 2011). The distinction between *action planning* (i.e. when, where, and how to act) and *coping planning* (i.e. how to overcome potential barriers) has been found to be useful in this regard (Sniehotta, Schwarzer, Scholz, & Schüz, 2005). Specific action plans contribute to behaviour initiation by driving the implementation of a new behaviour. In addition, the formulation of coping plans not only helps to deal with possible difficulties, but also increases the likelihood of long-term behaviour change (Sniehotta et al., 2005). Moreover, coping self-efficacy is relevant in the volitional phase and describes beliefs regarding one’s capability to deal with barriers that occur during the execution of the intended behaviour. This construct mediates the relationship between planning (action and coping planning) and behaviour. Once an action has been initiated (i.e. behaviour change), this behaviour is said to be attained. According to the HAPA, individuals can be classified in three groups: (1) non-intenders if they are in the motivational phase, (2) intenders in the volitional phase, and (3) actors when behaviour change has been attained.

### Consumers’ perception and behaviour related to antimicrobial resistance in food

Previous research has indicated that awareness regarding the transmission risk of antimicrobial-resistant bacteria through food is rather low among Swiss consumers (Lechner, Freivogel, Stärk, & Visschers, 2020). This lack of knowledge on foodborne illnesses caused by antimicrobial-resistant bacteria may be a barrier to implement hygienic practices during food handling (Angelillo, Foresta, Scozzafava, & Pavia, 2001; Taché & Carpentier, 2014). Therefore, it is essential to raise awareness and knowledge regarding the spread of antimicrobial-resistant bacteria through food products. In addition, consumers must learn about the antibacterial effects of different hygiene measures (i.e. positive outcome expectancies) because not everyone is familiar with them (Young & Waddell, 2016).

However, while providing information is necessary, it is insufficient to achieve a behaviour change (Corace & Garber, 2014; Kelly & Barker, 2016). Studies have indicated that there is no or little direct effect of knowledge on behaviour, but knowledge often influences the intention to act (Eggers, Aarø, Bos, Mathews, & de Vries, 2014). Moreover, individuals with the same level of knowledge of a hazard may estimate its riskiness differently (Ferrer & Klein, 2015). In particular, individuals who have not experienced food poisoning are more optimistic regarding the risk of improper food handling than those who have experienced food poisoning (Millman, Rigby, Edward-Jones, Lighton, & Jones, 2014). Similarly, belonging to certain high-risk groups increases an individual’s willingness to improve preventive behaviour when dealing with food (Young & Waddell, 2016). Thus, increased perceived personal risk might additionally motivate behaviour change (Hohman, Crano, & Niedbala, 2016). Further, it is important to reduce disagreement with recommendations for safe food handling because conflicting beliefs and perceptions hinder the implementation of preventive food-handling measures (Young & Waddell, 2016). The goal is that the associated benefits of food-handling behaviour will outweigh the perceived costs of implementing safe food-handling measures.

Moreover, research has indicated that self-efficacy is a strong predictor in explaining consumers’ intention for safe food handling (Freivogel & Visschers, 2020; Haapala & Probart, 2004), but there is also an intention-behaviour gap in food handling (Mullan et al., 2013). Coping planning has proved to support the transition from intention to behaviour, whereas action planning was—contrary to the predictions of the HAPA—not found to be a mediator between intention and food handling behaviour in previous research (Freivogel & Visschers, 2020; Mullan, Wong, & O'Moore, 2010). It must be noted that action planning may be irrelevant in this case because there is little planning freedom in food preparation; this is because the time point (morning, lunchtime, or evening) and location (kitchen) of meals are usually predetermined.

In the studies reported here, we investigated behaviour change techniques that aim to change determinants from both the motivation and volition phases. In fact, based on the literature review, we targeted the determinants knowledge, risk perception, positive outcome expectancy, coping self-efficacy, and planning. The goal of Study 1 was to strengthen individuals’ intention to adopt hygienic food-handling practices. We focused primarily on improving knowledge, positive outcome expectancies, and risk perception, which in turn are expected to strengthen intention. It did not seem worthwhile to focus on pre-intentional self-efficacy because this factor is generally high for food-handling measures (e.g. Freivogel & Visschers, 2020; Mullan et al., 2010). In Study 2, we focused on the determinants of the volitional phase. Therefore, we mainly targeted planning in order to bridge the intention-behaviour gap.

### Strategies to address the relevant determinants of behaviour change

Various frameworks suggest a number of strategies that are expected to bring about a change in the level of knowledge, positive outcome expectancies, risk perception, intention, and planning. We grounded our strategies on behaviour change techniques (BCT) taxonomies (Abraham & Michie, 2008; Kok et al., 2016; Michie et al., 2013). Each BCT prescribes the particular type of content as well as the type of determinant and the manner in which the former affects the latter. The strategies applied in our studies included BCTs aligned with either predictors of intention (Study 1) or with the determinants of the transition from mere intention to actual behaviour (Study 2).

In Study 1, our objective was to improve knowledge on antimicrobial-resistant bacteria in food and, therefore, provided factual information regarding this topic. Results from a previous study indicated that receiving factual information on what antimicrobials are, how bacteria become resistant, and the importance of prudent use of antimicrobials increases the general awareness regarding antimicrobial resistance (van Rijn, Haverkate, Achterberg, & Timen, 2019). However, whereas van Rijn et al. (2019) mainly relied on educating about antimicrobial resistance and antimicrobial treatments, we focused on the relevant information regarding food as transmission pathways of antimicrobial-resistant bacteria. Therefore, we developed an educational video describing the spread of antimicrobial resistance and the importance of safe food handling to enhance positive attitudes towards hygienic kitchen practices. The video included several BCTs: (1) *consciousness raising* (e.g. by providing information on health consequences), (2) *demonstrations of problem-solving behaviours*, and (3) *persuasive communication* (Abraham & Michie, 2008; Kok et al., 2016).

Videos have been successfully employed as a means of improving knowledge regarding health behaviour, such as applying sunscreen or condom use (Armstrong, Idriss, & Kim, 2011; Healton & Messeri, 1993; Sanderson & Yopyk, 2007). Compared to a pamphlet, videos were found to have significantly greater improvement in knowledge and preventive behaviour (Armstrong et al., 2011; Tuong, Larsen, & Armstrong, 2014).

As a BCT targeting risk perception, we applied *personalised messages* that included risk information on antimicrobial resistance in food (Harle, Downs, & Padman, 2012; Manuel, Abdulaziz, Perez, Beach, & Bennett, 2018; Mays et al., 2020). The personalised risk message was provided as printed text and a pictograph. The printed information enabled participants to set their own pace in reviewing information. Visual support, like pictographs, draw attention and enhance people’s understanding of complex risk messages (Dubé, Gagnon, & Vivion, 2020; Forrest, Sawyer, Hallowell, James, & Young, 2019; Hess, Visschers, & Siegrist, 2011; Visschers, Meertens, Passchier, & De Vries, 2009). The pictograph in our study is a portion of contaminated poultry meat in Switzerland.

In Study 2, we aimed to test a strategy to overcome the intention-behaviour gap. Therefore, we applied *goal setting* as BCT, which is a commonly used strategy in web-based interventions (Hou, Charlery, & Roberson, 2014) and has been documented to be an effective self-regulatory skill to promote preventive behaviour, like physical activity (Epton, Currie, & Armitage, 2017). The goal-setting theory assumes that planning arises after a person commits to a goal (Locke, Shaw, Saari, & Latham, 1981). In turn, planning is an important tool that supports the translation from intention to action. Goal setting is also addressed in the social cognitive theory (Bandura, 1991), which emphasises the importance of setting achievable goals as a means to increase self-efficacy, which can subsequently lead to behaviour change. Therefore, goal setting is more effective, particularly in terms of increasing self-efficacy, when individuals set specific, proximal, and moderately difficult goals (Bandura, 1991; Pearson, 2012). We combined goal setting with the BCT *planning coping responses* (i.e. consider how to overcome emerging barriers) followed by instructions on how to perform a certain behaviour.

Moreover, the BCT *framing* was used in both studies. Messages were mainly focused on the advantages of complying with safe food-handling behaviour, as previous research found support for the use of positive frames when targeting preventive behaviour (e.g. Gallagher & Updegraff, 2012). We included visual aids to improve attentional capture and understanding of the information (Antúnez, Giménez, Maiche, & Ares, 2015; Garcia-Retamero & Cokely, 2011).

### Aims of the present studies

In Study 1, we aimed to raise participants’ knowledge about antimicrobial resistance, risk perception of antimicrobial resistance and positive outcome expectancies on safe food-handling measures in order to increase their intention to adopt safe food handling. We developed the following two hypotheses in this regard:

1. Watching an educational video leads to an increase in knowledge about antimicrobial resistance, positive outcome expectancies, and intention as compared to watching a control video.
2. Receiving a personalised risk message regarding the prevalence of antimicrobial-resistant bacteria in poultry meat raises risk perception and intention compared to not receiving a personalised risk message.

In Study 2, we aimed to increase the prevalence of safe food-handling behaviour. We expected to achieve behaviour change by targeting predictors that mediate the intention-behaviour gap (i.e. the volitional phase in the HAPA). This led to the following hypothesis:

(3) Goal setting leads to an improvement in action planning, coping planning, coping self-efficacy, and safe food-handling behaviour compared to no goal setting at all.

Both studies were conducted online. The questionnaires were programmed in Questback (Questback, 2018). Ethical approval was granted for both studies from our institutional ethics committee (Reference number: EAaFE2019006).

## Study 1

### Method

#### Sample

Participants were members of an online survey panel operated by an internet marketing and research institute. German-speaking adults from Switzerland were eligible to participate. People who did not prepare raw poultry or red meat themselves were excluded from the study. A pre-stratification according to gender (50% female; 50% male) and age (33% 18-40yrs.; 42% 41-60yrs.; 25% 61-100yrs.) was applied in order to ensure that the sample was representative of the Swiss adult population (Swiss Statistics, 2019). The final study sample, after data cleaning, consisted of 328 food preparers. Power analysis was used a priori to determine the number of observations required to detect an expected small effect size which is based on previous related research (Sanderson & Yopyk, 2007). Power calculations for a MANOVA using G*Power (Faul, Erdfelder, Lang, & Buchner, 2007) indicated that a sample size of 289 was required to detect a small effect, *f*^2^ = .04 ($\eta_{p}^{2}$ = .04), maintaining the type I error at .05 and statistical power at .95.

#### Procedure

We set up a randomised control trial (RCT) with a 2 × 2 between factorial design in which video (antimicrobial resistance vs control) and personalised risk message (yes vs no) were between-subject factors. Participants were blinded about conditions. Predictors of the motivational HAPA phase (intention, risk perception, and positive and negative outcome expectancies) and knowledge were the dependent variables. The study was conducted online from October 2019 to November 2019. All participants were first presented a short explanation regarding the study procedure, without specifying the subject of the research. The introduction further emphasised the anonymous method of data collection and respondents were asked to provide their informed consent to participate in the study.

At the beginning of the questionnaire, demographics and meat consumption were assessed to verify an individual’s eligibility to participate. Participants were asked about their current safe food-handling behaviour in terms of how frequently they implemented different safe food-handling practices. Participants were then randomly assigned by the survey software to one of four conditions: (1) video on antimicrobial resistance with personalised risk message, (2) video on antimicrobial resistance without personalised risk message, (3) control video with personalised risk message, or (4) control video without personalised risk message (i.e. control condition). Respondents first watched one of the two videos and then read the personalised risk message (or not). Both before and after providing the BCTs, a question assessed the HAPA phase. Thereafter, participants were required to complete a questionnaire.

Participants were allowed to proceed with the questionnaire only if they answered all the questions. If they completed the survey, they received a small renumeration (redeemable points equivalent to 0.50 CHF) from the internet research company. On the last page, participants were debriefed on the research goal. Contact information and a link to the Federal Food Safety and Veterinary Office were presented in case participants were interested in more information about antimicrobial resistance or the preventive measures. Completing the survey took approximately 16 min.

#### Questionnaire

Items were adapted from previous studies (Chow & Mullan, 2010; Freivogel & Visschers, 2020; Kennedy et al., 2011). The questionnaire was pretested on a small sample (*n* = 3) to check the items’ comprehension. All items and their descriptive statistics can be found in the Appendix A (Table 1: items of HAPA variables; Table 3: knowledge items).

Preparation of meat, poultry, fish and seafood were each measured on a 6-response scale (1 = never to 6 = always). Both before and after providing the BCTs, a question assessed whether the participant was in the motivational, volitional or action phase (i.e. HAPA phase). Therefore, they first read the definition of safe food handling: ‘Safe food handling means that food is stored and prepared in such a way that pathogenic bacteria are killed or can’t spread around’. Then, they were asked to select which of the following three responses best represented their food-handling behaviour: ‘I do not intend to implement more safe food-handling measures’, ‘I intend to implement more safe food-handling measures’, or ‘I already implement safe food-handling measures’.

Next, six items evaluated self-reported food-handling behaviour (response scale: 1 = never to 6 = always). A series of sixteen items was designed to investigate participants’ *knowledge* regarding antimicrobial resistance and the spread of antimicrobial-resistant bacteria (response scale: true, false, I don’t know). *Risk perception* of unsafe food handling was assessed using thirteen items (response slider: 1 = unlikely to 10 = very likely), whereby nine items measured the susceptibility and four items the severity of an antimicrobial-resistant infection through food-handling. Negative and positive outcome expectancies of implementing preventive measures were each measured using five items (response slider: 1 = don’t agree at all to 10 = fully agree). The five items of *negative outcome expectancies* were designed to measure prevalent beliefs regarding the cons of safe food-handling among Swiss food preparers, like killing health-promoting bacteria. Items of *positive outcome expectancie*s measured participants’ beliefs regarding the antibacterial effect of food-handling measures.

Further, in order to identify random answers, we integrated the following control item: ‘This is a control question. Select ‘fully agree’, which corresponds to a 10 on the slider’. Participants who did not answer this item correctly were excluded from all analyses. We assessed participants’ *intention* to conduct each food-handling measure using six items (response slider: 1 = don’t agree at all to 10 = fully agree).

Participants were further asked to *evaluate the information received* on semantic differential scales (6-point scales on informative, comprehensive, important, alarming, convincing, motivating, realistic, relevant, and sufficient)*.* At the end of the questionnaire, the level of education and household composition were collected from all participants.

#### Behaviour change techniques

The *video on antimicrobial resistance* was based on cartoons and used a main character to explain how antimicrobial-resistant bacteria can be spread through animals. The video^1^ on antimicrobial resistance targeted knowledge gaps and misconceptions among Swiss consumers found in a prior study (e.g. the misbeliefs that organic meat is not contaminated with antimicrobial-resistant bacteria and that carriers of antimicrobial-resistant bacteria always show symptoms) (Lechner et al., 2020). The first scene explained the emergence of antimicrobial-resistant bacteria before illustrating the transmission risk between humans and animals. The antimicrobial-resistant bacteria were presented as red and spiny. Thereafter, the increase in the number of cases with antimicrobial-resistant infections was emphasised. Next, a chicken was shown to demonstrate how animal food products can be contaminated with antimicrobial-resistant bacteria. At the end of the video, the preventive measures of thoroughly washing, cooking, separating, and chilling were recommended. The topic of the *control video* was human decision-making; the video explained the differences between gut feelings and deliberative thinking. The duration of both videos was approximately two minutes. Participants were allowed to watch the video several times.

The *personalised risk message* included an illustration depicting ten chicken legs, where six out of ten were contaminated with bacteria (see Appendix C). In addition, a message about the prevalence of antimicrobial-resistant bacteria on Swiss poultry meat and the high consumption of poultry meat in Switzerland was provided. In Switzerland, poultry represents the highest exposure risk to antimicrobial resistance for consumers (Collineau et al., 2018; Jans et al., 2018) and is the second most consumed meat after pork (Proviande, 2017). The message referred to the respondent’s own poultry consumption, which was measured at the beginning of the survey (e.g. ‘According to your answer, you also prepare raw poultry *once a day [several times a week, once a week, several times a month, or once a month]*. Please store and prepare food hygienically to consume it safely’).

#### Data analyses

**Figure 1.**
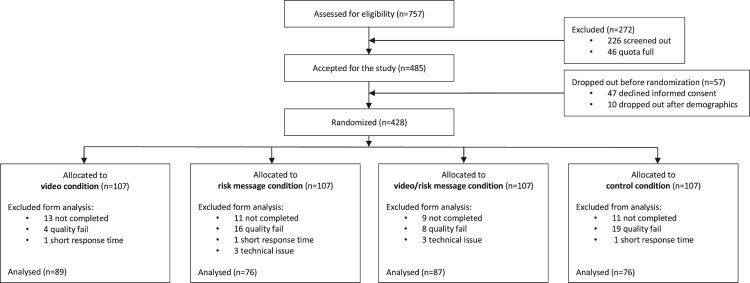
Flow diagram of Study 1.

Data analyses were conducted with SPSS 25 (IBM Corp., 2017) and R (R Core Team, 2020). Only respondents who completed all items were included. After the exclusion of subjects with a short response time (threshold: less than half of the median time) and subjects who did not correctly respond to the control question (see Figure 1), 328 valid respondents remained in the data set.

As preliminary data analyses, we applied several scale validation analyses (Dima, 2018). After reviewing descriptive statistics at item level, we conducted Mokken scale analyses (MSA) to check for homogeneity of the items and scales. MSA were performed using the *mokken* package in R (Van der Ark, 2007, 2012). The *aisp* function in the *mokken* package was used to identify potential Mokken or latent unidimensional scales. One MSA was conducted with the items of the HAPA variables and another with the knowledge items. Each of the subscales were then evaluated for unidimensionality, monotonicity, and invariant item ordering (IIO). We discarded items when *Crit* value was above 80 (Van Schuur, 2003).

For the HAPA-items, the AISP analysis suggested four unidimensional subscales, which were overall similar to the HAPA constructs (see Appendix A, Table 2). Also, four challenging HAPA items were identified, which were excluded from further analyses (Sijtsma & Molenaar, 2002). Although Mokken values suggested one scale for intention and behaviour, we evaluated them separately because theory suggests two different constructs. Moreover, various preventive measures were assessed and therefore some heterogeneity was to be expected.

For the knowledge items, incorrect and ‘I do not know’ answers were merged together before they entered the MSA. The second MSA indicated that the knowledge scale was divided into three subscales, whereby one subscale mainly measured knowledge about antimicrobial resistance in general (Appendix A, Table 3). The second subscale mainly measured knowledge about health risks caused by resistant bacteria, whereas the third subscale measured knowledge about the transmission of resistant bacteria via food.

Next, exploratory factor analysis (EFA) was performed on the HAPA items to test their construct validity. The outcomes of the EFA confirmed the hypothesised HAPA constructs, except for risk perception. The latter was split into two constructs: *perceived susceptibility* and *perceived severity*, which is in line with theory (e.g. Schwarzer, 2016). Intention was remained as a single construct, although the items loaded on two separate factors. It is not surprising that the intention items loaded on different factors because they covered various food-handling behaviours. The same applies to the behaviour items. Cronbach's alpha was then calculated to test each construct’s internal consistency (see Appendix A, Table 2). We did not exclude additional items, based on the reliability analysis, because it would not have considerably improved the internal reliability of the construct and the items were necessary to include in the construct based on its underlying theory.

Subsequently, we calculated the mean score per construct over all respective items for each respondent and used these scores in all further analyses. The number of correct knowledge items per respondent were summed up (ranging between 0 and 8 for general knowledge; 0 and 4 for knowledge about health risks; 0 and 3 for knowledge about transmission). Descriptive statistics were then applied to describe food-handling behaviour and the other variables.

The effect of the BCTs on the knowledge subscales and the HAPA variables perceived susceptibility, perceived severity, positive and negative outcome expectancies, and intention were investigated in two separate multivariate analyses of variance (MANOVA). A univariate analysis of variance (ANOVA) was performed to determine if the evaluation of the received information differed due to the type of video and whether participants received a personalised risk message. Initially, the homogeneity of variance was verified. (M)ANOVA is robust against the violation of normality with equal sample sizes for each condition (Huberty & Olejnik, 2006). The MANOVA is also robust against violations of homogeneity of variance-covariance matrices assumption, if group sizes are over 30 (Allen & Bennett, 2008). The significance level was set at α = .025 to evaluate the outcomes of both MANOVAs and the ANOVA, because the homogeneity of variances was violated for some of the dependent variables^2^ (Tabachnick, Fidell, & Ullman, 2007).

Last, a chi-square test was employed to examine the HAPA phases (motivational, volitional, or action) between the conditions before and after the BCTs. Since the chi-square test for independence cannot be applied in a repeated measures design, Friedman’s tests were conducted to compare the HAPA phases before and after receiving the BCTs for each condition. Bonferroni correction adjusted the statistical significance to α = .025, in order to reduce the probability of a Type I error. Frequency distributions, means, and standard deviations were used to describe the demographic and background characteristics of participants.

## Results

### Demographic characteristics

The demographic sample characteristics are presented in Table 1. The age range was 18–84 years. Demographic characteristics (i.e. gender, age, education level and household composition) did not differ among conditions, but there were differences in fish consumption (Table 1).^3^

**Table 1.** Demographic characteristics of the sample of Study 1 in total and per condition, including statistical tests examining possible differences between conditions.

	Total *N* = 328		Video (*n* = 89)		Risk Message (*n* = 76)		Risk Message/Video (*n* = 87)		Control (*n* = 76)			
	*N*	%	*n*	%	*N*	%	*n*	%	*n*	%	χ^2^	*p*
*Gender*											4.86	.18
Male	166	50.6	48	53.9	45	59.2	39	44.8	34	44.7		
Female	162	49.4	41	46.1	31	40.8	48	55.2	42	55.3		
*Education level*											7.10	.31
Primary or secondary school	17	5.2	4	4.5	2	2.6	8	9.2	3	3.9		
Vocational or higher secondary school	224	68.3	63	70.8	56	73.7	51	58.6	54	71.1		
College/University degree	87	26.5	22	24.7	18	23.7	28	32.2	19	25.0		
*Household composition*											9.48	.39
Single-person household	73	22.2	16	18.0	19	25.0	21	24.1	17	22.4		
With partner	126	38.4	37	41.6	31	40.8	27	31.0	31	40.8		
With children	96	29.3	23	25.8	22	28.9	32	36.8	19	25.0		
Another household composition	33	10.1	13	14.6	4	5.3	7	8.1	9	11.8		
	*M*	(*SD*)	*M*	(*SD*)	*M*	(*SD*)	*M*	(*SD*)	*M*	(*SD*)	*F*	*p*
Age	49.43	(15.28)	47.93	(15.16)	50.08	(14.61)	48.54	(14.12)	51.54	(17.06)	.92	.43
*Frequency of preparing … *												
Raw red meat	4.02	(1.13)	3.91	(1.25)	4.13	(1.06)	4.07	(1.08)	4.00	(1.12)	.59	.63
Raw poultry	3.59	(1.02)	3.57	(0.96)	3.74	(1.09)	3.62	(1.03)	3.45	(1.01)	1.05	.37
Raw fish	2.44	(1.22)	2.47	(1.10)	2.74	(1.34)	2.21	(1.16)	2.36	(1.23)	2.77	.04*
Raw seafood	1.50	(0.87)	1.54	(0.93)	1.54	(0.93)	1.47	(0.87)	1.43	(0.72)	.29	.83

Notes: * *p* < .05. Video indicates the group received the video on antimicrobial resistance. Risk Message indicates that the group received a personalised risk message.

### Descriptive data

Overall, participants reported handling their food rather hygienically (see Table 2, behaviour)*.* Chilling food properly was implemented the most often (*M* = 5.65*, SD* *=* 0.75), while using separate cutting boards was implemented the least (*M* = 4.31*, SD* *=* 1.75). In line with HAPA, perceived susceptibility and perceived severity and outcome expectancies significantly correlated with intention and intention correlated with behaviour.

**Table 2.** Descriptive statistics and correlations among variables in Study 1.

	Variable	*M* (*SD*)	1	2	3	4	5	6	7	8
1.	Behaviour	5.21 (0.79)	–							
2.	Intention	9.17 (1.08)	.54**	–						
3.	Susceptibility for Risk	6.08 (1. 78)	.04	.14**	–					
4.	Severity of Risk	7.68 (1.66)	.13*	.32**	.49**	–				
5.	Positive Outcome Expectancy	8.36 (1.30)	.24**	.47**	.15**	.23**	–			
6.	Negative Outcome Expectancy	3.02 (1.62)	−.21**	−.30**	−.02	−.04	−.14 *	–		
7.	General Knowledge	5.57 (1.76)	−.00	.11	.21**	.12*	.13*	−.17**	–	
8	Knowledge about Health Risks	3.28 (0.92)	.12*	.15**	.17**	.15**	.28**	−.10	.32**	–
9	Knowledge about Transmission	2.19 (0.79)	−.00	−.01	.16**	.04	.11*	.01	.14*	.32**

Notes. * *p* < .05, ** *p* < .01. Response scales: behaviour (1-6), intention (1-10), perceived susceptibility and severity of risk (1-10), positive and negative outcome expectancy (1-10), knowledge subscales: general (0-8); health risks (0-4), transmission (0-3).

Before receiving the BCTs, 10.7% (*n* = 35) of the participants were found to be non-intenders, 15.5% (*n* = 51) intenders, and 242 (73.8%) actors. After the BCTs, the proportion of intenders increased to 20.7% (*n* = 68). The majority still considered themselves as actors (*n* = 236, 72%), while 7.3% (*n* = 24) remained non-intenders.

### Impact of the behaviour change techniques

**Figure 2.**
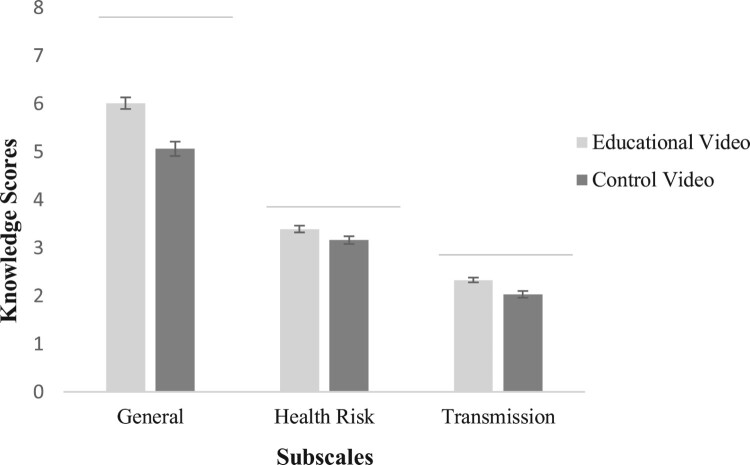
Mean scores (and SEs) of knowledge about antimicrobial resistance in general, about health risk and about transmission among participants who received the educational video and those who received the control video. Notes. The number of correct knowledge items per subscale were summed up. The maximum score of each subscale is indicated by a grey line. Response scales: general knowledge (0-8), knowledge about health risks (0-4) and knowledge about transmission (0-3).

The results of the MANOVA on the knowledge subscales indicated a significant main effect for video, *F*(3, 322) = 11.34, *p* = .001, $\eta_{p}^{2}$ = .10, but not for risk message, *F*(3, 322) = 0.94, *p* = .42. There was no interaction effect of video and risk message on any of the knowledge scales, *F*(3, 322) = 0.35, *p* = .79. The univariate comparisons indicated a significant main effect of video on general knowledge about antimicrobial resistance, *F*(3, 324) = 25.25, *p* = .001, $\eta_{p}^{2}$ = .07. Figure 2 illustrates that knowledge on antimicrobial resistance was significantly higher among participants who watched the educational video on antimicrobial resistance (*M* = 6.01, *SE* = .12) than among participants who watched the control video (*M* = 5.06, *SE* = .15). There was also a significant main effect of video on knowledge about health risks, *F*(1, 324) = 5.32, *p* = .02, $\eta_{p}^{2}$ = .02, whereby knowledge about health risks caused by antimicrobial-resistant bacteria was higher among participants who watched the educational video (*M* = 3.39, *SE* = .07) than among participants who watched the control video (*M* = 3.16, *SE* = .08). The significant main effect of video on knowledge about transmission, *F*(1, 324) = 11.90, *p* = .001, $\eta_{p}^{2}$ = .04, further indicated that knowledge about the transmission of antimicrobial-resistant bacteria via food was higher among participants who watched the educational video (*M* = 2.33, *SE* = .05) than among participants who watched the control video (*M* = 2.03, *SE* = .07).

The second MANOVA indicated significant multivariate effects of video, *F*(5, 320) = 3.96, *p* = .001, $\eta_{p}^{2}$ = .06, and of personalised risk message, *F*(5, 320) = 3.10, *p* = .01, $\eta_{p}^{2}$ = .05, on all dependent variables. There was no significant multivariate interaction between video and personalised risk message on the dependent variables, *F*(5, 320) = 0.52, *p* = .76. The univariate comparisons indicated a significant main effect of video on perceived susceptibility, *F*(1, 324) = 17.68, *p* = .001, $\eta_{p}^{2}$ = .05. Respondents who had watched the educational video perceived a contamination with antimicrobial-resistant bacteria through food as more likely (*M* = 6.45, *SE* = .13) than those who had watched the control video (*M* = 5.65, *SE* = .14). There were no significant main effects of video on intention, perceived severity, positive outcome expectancies, or negative outcome expectancies, *F*s < 1.96, *p*s > .19.

In addition, the univariate comparisons indicated a marginally significant main effect of personalised risk message on positive outcome expectancies, *F*(1, 324) = 5.00, *p* = .03, $\eta_{p}^{2}$ = .02 (α = .025). Respondents who had received the personalised risk message anticipated more positive consequences as a result of safe food-handling (*M* = 8.24, *SE* = .09) than those who had not received the personalised risk message (*M* = 7.89, *SE* = .10). There were no significant main effects of personalised risk message on intention, negative outcome expectancies, perceived susceptibility of risk or perceived severity of risk, *F*s < 3.70, *p*s > .06.

**Figure 3.**
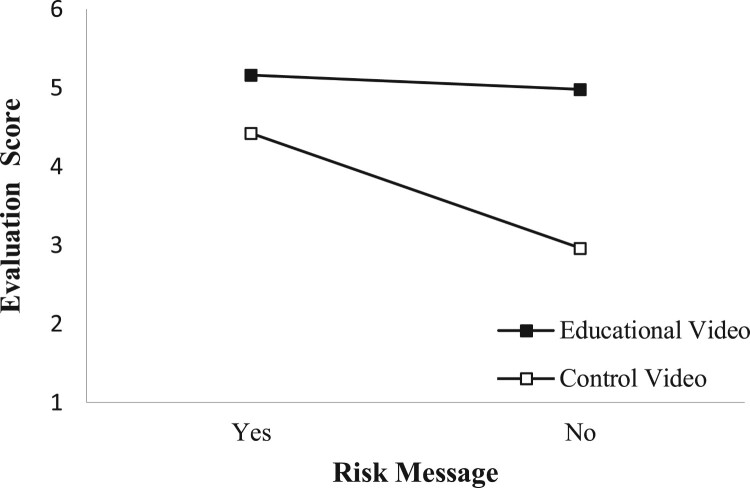
Mean information evaluation scores per video condition and risk message condition.

The ANOVA on evaluation of the received information revealed that participants who watched the educational video evaluated the received information significantly more positively (*M* = 5.07, *SE* = .06) than participants who watched the control video (*M* = 3.69, *SE* = .10), *F*(1, 324) = 197.97, *p* < .001, $\eta_{p}^{2}$ = .38. Further, participants who received the personalised risk message also evaluated the received information significantly more positively (*M* = 4.81, *SE* = .07) than participants without the personalised risk message (*M* = 4.05, *SE* = .11), *F*(1, 324) = 69.95, *p* < .001, $\eta_{p}^{2}$ = .18. The interaction between video and risk message was also significant, *F*(1, 324) = 42.47, *p* < .001, $\eta_{p}^{2}$ = .12. As depicted in Figure 3, participants who received the control video without a personalised risk message (*M* = 2.96, *SE* = .13) evaluated the information more negatively than those who received the control video with the personalised risk message (*M* = 4.42, *SE* = .10) and those who received the educational video with the personalised risk message (*M* = 5.16, *SE* = .07) or without the personalised risk message (*M* = 4.98, *SE* = .08).

There was no difference in terms of the HAPA phases between the four conditions before receiving the BCTs, χ^2^(6) = 3.76, *p* = .71. Another chi-square test of independence revealed that there was no significant association between condition and attained HAPA phase after receiving the BCTs, χ^2^(6) = 7.89, *p* = .25. Further, Friedman’s ANOVA tests found no significant change in the respondents’ presence in the HAPA phases before and after receiving the BCTs in any of the four conditions, χ^2^s < .60, *ps* > .44.

## Study 2

### Method

#### Sample

**Figure 4.**
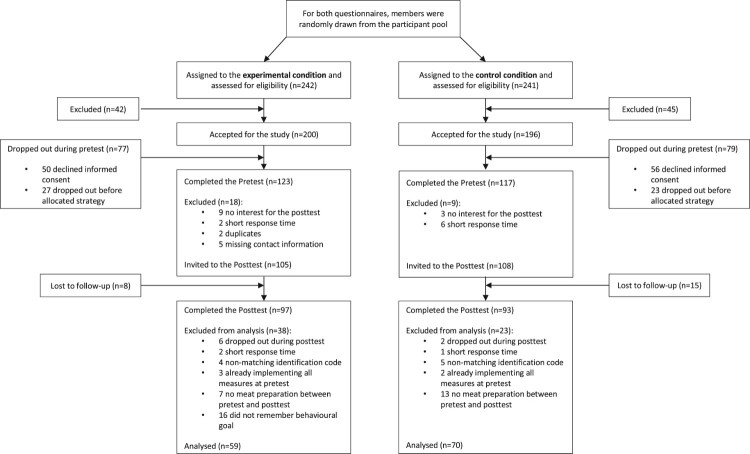
Flow diagram of Study 2.

Participants were members of a participant pool from the Psychological Institute of the University of Zurich. Only adult members who occasionally prepared poultry or red meat themselves were eligible to participate. The sample was not representative of the Swiss adult population (see Table 3) since the majority of the sample was female (Swiss Statistics, 2019). Moreover, most of the participants were students and therefore highly educated. After data cleaning, the final study sample consisted of 129 respondents (see Figure 4). The follow-up rate was 89.1%. Power calculations for the two ANOVAs using G*Power indicated that a sample size of 158 was required to detect a small effect (*f*^2^ =  .25; $\eta_{p}^{2}$ = .02). The expected effect size was based on previous research (Webb, Joseph, Yardley, & Michie, 2010). Type I error was set at .05 and statistical power at .95.

**Table 3.** Demographic characteristics of the sample of Study 2 in total and per condition.

	Total Sample		Experimental (*n* = 59)		Control (*n* = 70)			
	*N*	(%)	*n*	(%)	*n*	(%)	*χ*^2^	*p*
*Gender*							.39	.53
Males	33	(25.6)	13	(22.0)	20	(28.6)		
Females	96	(74.4)	46	(78.0)	50	(71.4)		
*Education level*							3.66	.16
Primary or secondary school	3	(2.3)	3	(5.1)	0	(0)		
Vocational or higher secondary school	39	(30.2)	17	(28.8)	22	(31.4)		
University degree	87	(67.5)	39	(66.1)	48	(68.6)		
*Household composition*							4.73	.19
Single-person household	19	(14.7)	9	(15.3)	10	(14.3)		
With partner	49	(38.0)	22	(37.3)	27	(38.6)		
With children	19	(14.7)	5	(8.5)	14	(20.0)		
With parents	19	(14.7)	8	(13.6)	11	(15.7)		
Another household composition	23	(17.9)	15	(25.3)	8	(11.4)		
	*M*	*(SD)*	*M*	*(SD)*	*M*	*(SD)*	*F*	*p*
Age	35.52	(14.16)	32.71	(12.30)	37.89	(15.25)	4.38	.04*
*Frequency of preparing … *								
Raw red meat	3.81	(1.13)	3.63	(1.30)	3.97	(0.95)	3.01	.09
Raw poultry	3.56	(1.05)	3.54	(1.10)	3.57	(1.00)	.03	.88
Raw fish	2.15	(1.37)	2.07	(1.03)	2.21	(0.98)	.68	.41
Raw seafood	1.37	(0.76)	1.37	(0.81)	1.37	(0.73)	.00	.99

* *p* < .05.

#### Procedure

Study 2 was conducted from January to February 2020. We employed a RCT with a 2 × 2 factorial design in which planning (goal setting vs. control) was a between-subjects factor and the measurement point (pre- vs. posttest) a within-subject factor. The posttest began 10 days after the pretest. Participants’ food-handling, coping self-efficacy, planning of implementing preventive measures, and planning of overcoming emerging barriers were the dependent variables.

We randomly selected two groups of members from the participant panel. The first group received the invitation for the experimental condition and the other group received the invitation for the control condition. Thus, randomisation occurred before panel members were approached. Participants were blinded about the condition and received an e-mail invitation to join a study on the handling of food. Participants were eligible to win one of six retail store vouchers worth 25 CHF or 50 CHF as incentive after completing the pretest and posttest, respectively. At the pretest stage, all participants were presented a brief introduction to the study, including a short explanation on the study procedure and the two measurement points. The introduction further emphasised the anonymous method of data collection and processing.

After providing informed consent and verifying the inclusion criteria, we assessed participants’ HAPA phase regarding safe food-handling. Subsequently, self-reported food-handling behaviour was measured, and participants were required to read a short text on the spread of antimicrobial-resistant bacteria through food (see Appendix D). Participants in the *experimental condition* selected one preventive measure that they planned to implement during the following 10 days while preparing raw meat, fish, or seafood (i.e. goal setting). They were invited to anticipate potential barriers and consider how these barriers could be overcome before receiving advice on how to implement the selected goal. Participants in the *control condition* did not receive a BCT.

At the end of the pretest, all participants evaluated the received information, whereby the control group only answered questions on the text about antimicrobial resistance and the experimental group additionally evaluated the advice regarding their selected goal. Finally, participants were reminded to implement their selected food-handling goal (experimental condition) or to keep an eye on hygienic food-handling in general (control condition) in the next 10 days. Further, participants were asked to provide their e-mail addresses in order to receive the invitation for the posttest and to participate in the raffle of the vouchers. E-mail addresses were collected and stored separately from their questionnaire data. Further, an anonymous four-digit easy-to-remember identification code generated by participants themselves enabled us to match the data from the two measurements.

Ten days after completing the pretest, participants received an invitation for the posttest by email. The survey assessed respondents’ food-handling behaviour over the last 10 days, coping self-efficacy, and planning for the implementation of preventive measures and overcoming emerging barriers. At the end of the questionnaire, the level of education and household composition were collected from all participants. The completion of the pretest took approximately 9 min and of the posttest approximately 14 min.

#### Questionnaire

Items were mostly adapted from previous studies and translated into German (Chow & Mullan, 2010). Pretesting of the questionnaire was done on a small sample (*n* = 3) to check the items’ comprehension. The same question as that in Study 1 assessed whether the participant was in the motivational, volitional, or action phase (HAPA phase).

Items of HAPA variables and their descriptive statistics can be found in the Appendix B, Table 1. Self-reported *food-handling behaviour* was measured using seven items (response scale: 1 = never to 6 = always). The food-handling measures in Study 2 partially differed from those in Study 1 because measures with high mean values (i.e. indicating high implementation and little improvement potential) in the first study were replaced in the second study. We made sure that measures for all four core practices of food safety – washing, cooking, separating and chilling– remained in the questionnaire (Peters, 2018). Otherwise, measures with a mean behaviour frequency > 5 were excluded or merged with a related measure.

Further, the *awareness* of antimicrobial resistance in food was assessed on a slider, preceded by the question, ‘How likely do you think it is that antibiotic resistant bacteria will spread through food?’ (response slider: 1 = unlikely to 10 = very likely). A set of five items measured coping self-efficacy (response scale: 1 = don’t agree at all to 6 = fully agree). Planning was measured using three items (response scale: 1 = don’t agree at all to 6 = fully agree): Two items for *action planning* and one item for *coping planning*. As in Study 1, the received information was evaluated on semantic differential scales (6-point scales for informative, comprehensible, important, alarming, convincing, motivational, realistic, relevant, and sufficient).

#### Behaviour change techniques

Participants in both conditions were provided with information on antimicrobial resistance (see Appendix D). In the experimental condition (i.e. goal setting), individuals were asked to select one of seven goals to work on in the coming 10 days and received advice on how to achieve their selected goal. Each piece of advice included an illustration and a short explanatory text (see Appendix E). Participants in the control condition received no further information or instructions.

#### Data analyses

SPSS 25 (IBM Corp., 2017) and R (R Core Team, 2020) were again used for data cleaning and analyses. Cases with missing data, non-matching codes, duplicate data, or an implausibly short amount of time on the questionnaires (less than half of the median) were eliminated. From the remaining data set, we also excluded data of participants who had already implemented all safe food-handling measures during the pretest; of participants who did not prepare raw meat, poultry, fish, or seafood between pretest and posttest; and of participants from the experimental condition who did not remember their determined goal correctly (see Figure 4). In the final sample, 129 respondents were included.

As in Study 1, scale validation analyses were conducted (Dima, 2018). Descriptive statistics on item level, MSA, EFA and Cronbach's alpha were calculated with the HAPA variables at pretest and posttest separately. The AISP analysis suggested two unidimensional subscales for the items at pretest and one unidimensional scale for the items at posttest. Following the HAPA, we evaluated the scales coping self-efficacy, behaviour and planning separately for unidimensionality, monotonicity, and IIO. Although the results of these analyses did not support a unidimensional behaviour scale, no items were excluded from this scale because we wanted to assess a broad range of preventive measures. Moreover, we separated the action planning and coping planning items based on theory. The coping self-efficacy item with a high factor loading on the behaviour factor at posttest was not excluded from further analysis because it loaded on the expected factor at pretest. All results are reported in the Appendix B, Table 2 and Table 3.

Subsequently, we calculated the mean score per construct over all respective variables per respondent and used these in all further analyses. Descriptive statistics were calculated for all variables. The effects of goal setting on behaviour and coping self-efficacy were analysed with repeated-measures ANOVAs. The between-groups factor was conditions (control vs. experimental) and the within-subjects factor was time-point (pretest vs. posttest). Bonferroni correction was used to prevent alpha inflation. Assumptions for a 2 × 2 mixed repeated-measures ANOVA were verified. Therefore, the significance level was set at α = .025 for behaviour and at α = .01 for coping self-efficacy since the equality of variances was violated for the latter (Tabachnick et al., 2007). Bonferroni-corrected post-hoc tests were employed to further analyse significant interaction effects. In addition, one-way ANCOVA was performed to determine the significant differences between the two conditions regarding coping planning at posttest, controlling for age.^4^ Further, an independent t-test was used to compare differences in action planning between the two conditions at posttest. The same statistical procedures were applied in Study 2 as those in Study 1 in order to compare the number of respondents in the HAPA phases between the two conditions of pretest and posttest.

## Results

### Demographic characteristics

The majority of participants were females and under 40 years of age. Demographic sample characteristics are presented in Table 3.

### Descriptive data

In general, the implementation of safe food-handling behaviour was high during pretest (*M* = 4.76, *SD* = 0.71). The descriptives of each of the seven food-handling measures are presented in Appendix B, Table 1. Mean awareness about antimicrobial resistance in food was 6.91 (*SD* = 2.10). The mean score for the evaluation of the information regarding antimicrobial resistance was rather high (*M* = 4.70, *SD* = 0.79). The mean evaluation of the information on preventive measures by the respondents in the experimental condition was similarly high (*M* = 4.82, *SD* = 0.74). Participants had also a high level of baseline coping self-efficacy (*M* = 4.81, *SD* = 0.95).

The variables were found to be highly correlated between time points (see Table 4). Moreover, the correlations between the variables were all in the expected direction. There was no significant positive association between awareness for antimicrobial resistance and behaviour. Correlation between age and the dependent variables were calculated because age differed between the two conditions (see Table 3).^5^

**Table 4.** Correlations among the variables at pre- and posttest in Study 2.

	Variable	1	2	3	4	5	6	7
	*Pretest*							
1.	Behaviour	–						
2.	Self-efficacy	.47**	–					
3.	Awareness	.15	.30**	–				
	*Posttest*							
4.	Behaviour	.74**	.50**	.11	–			
5.	Self-efficacy	.50**	.67**	.22*	.56**	–		
6.	Coping planning	.41**	.30*	.20*	.49**	.49**	–	
7.	Action planning	.41**	.37**	.22*	.56**	.55**	.78**	
8.	Age	.07	.09	−.15	.04	.12	.24**	.09

Notes. * *p* < .05, ** p < .01. Response scales: behaviour (1-6), coping self-efficacy (1-6), awareness for antimicrobial resistance (1-10), coping planning and action planning (1-6).

At pretest, 14.8% (*n* = 22) of participants were in the motivational phase of the HAPA, 10.1% (*n* = 15) in the volitional phase and the majority believed to handle food hygienically already (i.e. action phase, *n* = 112, 75.2%). Ten days later, at posttest, the percentage of intenders increased to 23.5% (*n* = 35), whereas the percentage of actors decreased to 63.8% (*n* = 95); the minority remained non-intenders (*n* = 19, 12.8%).

### Impact of the behaviour change techniques

The results of the repeated measures ANOVA revealed that there was no significant main effect of time, *F* (1, 127)  =  0.67, *p*  =  .41, and no significant main effect of condition on participants` behaviour, *F* (1, 127)  =  .17, *p*  =  .68. In contrast, there was a significant condition*time point interaction effect on behaviour, *F* (1, 127)  =  5.64, *p*  =  .02, $\eta_{p}^{2}$ =  .04, thereby indicating that participants in the goal-setting condition implemented more food-handling measures at posttest than pretest compared to those in the control condition (Table 5).

**Table 5.** Means and standard errors of food-handling behaviour and coping self-efficacy in both conditions and at both time points, as well as the difference between time points, Study 2.

Variable/	Pretest		Posttest		ΔTime points	
Condition	*M*	(*SE*)	*M*	*(SE)*	*M*	*(SE)*
*Behaviour*						
Experimental	4.72	(.09)	4.88	(.10)	−0.16	(.08)
Control	4.79	(.08)	4.71	(.10)	−0.08	(.06)
*Self-Efficacy*						
Experimental	4.74	(.11)	4.66	(.12)	−0.08	(.11)
Control	4.86	(.13)	4.62	(.14)	−0.25	(.10)
*Action Planning*						
Experimental			4.20	(.18)		
Control			3.64	(.19)		
*Coping Planning*						
Experimental			3.78	(.18)		
Control			3.27	(.20)		

A second ANOVA indicated neither a statistically significant difference between time points, *F*(1, 127) = 5.02, *p* = .03, nor between the goal-setting and control conditions in terms of coping self-efficacy, *F*(1, 127)  =  .07, *p*  =  .80. In addition, no significant condition*time point interaction was found for coping self-efficacy, *F*(1, 127)  =  1.19, *p*  =  .28.

A one-way ANCOVA revealed a significant effect of condition on coping planning after controlling for age, *F*(1, 126) = 6.04, *p* = .02, $\eta_{p}^{2}$ = .05. Participants in the goal-setting condition planned more regarding how to overcome barriers (*M_adj_* = 3.87, *SE* = .20) compared to participants in the control condition (*M_adj_* = 3.20, *SE* = .18). The t-test indicated that participants in the experimental condition showed significantly higher action planning for safe food-handling measures than participants in the control condition (Table 5), *t*(127) = 2.12, *p* = .04, *d* = .38.

The chi-square test of independence indicated no significant relationship between condition and the attained HAPA phase at pretest, χ*^2^*(2) = 1.13, *p* = .57. However, at posttest, we found a significant association between condition and attained HAPA phase, χ^2^(2) = 8.30, *p* = .02. In other words, the control condition included as many intenders (*n* = 13, 18.6%) as non-intenders (*n* = 13, 18.6%) as well as 44 (62.9%) actors. In the experimental condition, there were fewer non-intenders (*n* = 2, 3.4%) and more intenders (*n* = 18, 30.5%) and actors (*n* = 39, 66.1%) than in the control condition. However, the Friedman test found no statistically significant difference between the HAPA phases at pretest and posttest in the experimental or control conditions, χ^2^s(1) < 1.14, *p*s > .29.

## Discussion

The two studies in this paper tested whether BCTs targeting pre-intentional HAPA determinants would increase consumers’ intention to engage in more hygienic food-handling behaviours and whether BCTs targeting post-intentional HAPA determinants would increase consumers’ implementation of hygienic food-handling behaviour. Study 1 evaluated the usefulness of an educational video with respect to improved knowledge and risk perception regarding the transmission risk of antimicrobial-resistant bacteria through food. The first hypothesis is partially confirmed. Our findings indicated that consciousness raised by providing information on the risk of antimicrobial-resistant bacteria among Swiss consumers. Watching the educational video led to an increase in knowledge about resistance compared to watching the control video. However, the effect of the video differed according to the specific knowledge measured. The result for knowledge about health risks was not highly significant as it was for general knowledge or knowledge about transmission. Although the BCTs included in the educational video did not explicitly target risk perception, the perceived susceptibility for the transmission of antimicrobial-resistant bacteria increased after watching the educational video. We found that information regarding the risk source is sufficient to strengthen the belief about the occurrence of antimicrobial resistance in food. The content of this video succeeded in persuading people that the spread of antimicrobial-resistant bacteria through food is a personal risk. Compared to positive outcome expectancies and perceived severity, perceived susceptibility was low in the studied sample. Further, contrary to expectations, the provision of problem-solving behaviour at the end of the educational video could not enhance positive outcome expectancies. According to the literature, raising awareness must be quickly followed by an increase in problem-solving ability (Abraham & Michie, 2008). Apparently, merely demonstrating the preventive behaviour was not sufficient to increase positive outcome expectancies. According to the Model of Persuasion (McGuire, 1968), an audience believes a message only after understanding the value or content of the new information. Since not all consumers are aware of the antibacterial effect of hygienic food-handling measures (Lechner et al., 2020), our respondents may not have understood the association between the various measures related to washing, cooking, separating, and chilling (e.g. cooking kills bacteria and chilling stops bacterial growth). Therefore, a transition to acceptance of the message in the educational video may have been unlikely, because participants did not understand the relevance of the different measures as being effective measures against antimicrobial-resistant bacteria. Alternatively, participants may not have paid sufficient attention to the video content because of a lack of cognitive capacity. The preventive measures were presented at the end of the educational video, after other information had already been processed. Therefore, it is possible that participants had not yet been able to process and comprehend the message on preventive behaviour. Further, even more attentional resources are required when the information is not perceived as self-relevant (Bargh, 1982). The fact that the optimism bias (Weinstein, 1989) has been found to explain consumers’ low risk perception of foodborne illnesses due to their own food preparation (Fischer & Frewer, 2008) and their low risk perception of transmission of antimicrobial-resistant bacteria during food preparation (Lechner et al., 2020) indicates that participants may not have considered the information from the video as being personally relevant. They may have believed themselves to be safer cooks in comparison to other people. Another possible explanation for the missing increase in positive outcome expectancies after watching the educational video might be that the preventive effect of the recommended behaviour—that is, the reduction of antimicrobial-resistant bacteria—is not detectable by consumers and could, therefore, not have a positive effect on outcome expectancies. Most participants reported that they were already implementing a few of the measures (e.g. washing hands after the contact with raw meat). Not experiencing any benefits from previously implemented measures could have influenced their expectations for similar behaviour. Participants may only have perceived the associated costs of this behaviour, which outweigh the perceived benefits. Thus, we propose that further research must present both sides (pros and cons) of implementing the behaviour since this type of message may be viewed as more credible (Keller & Lehmann, 2008).

Contrary to the educational video, the personalised risk message could neither affect perceived susceptibility nor perceived severity. Therefore, our second hypothesis was not confirmed. However, contrary to expectations, the positive outcome expectancy marginally increased after receiving the risk message. It seems that participants connected a reduction of the bacterial load provided in the risk message with the food-handling measures assessed in the questionnaire.

Receiving the personalised risk message did not lead to an increase in risk perception and intention as compared to not receiving such a message. Personal experience is a factor that strongly influences risk perception (Ohman, 2017). It is possible that referring to poultry preparation as a risk factor did not draw on participants’ subjective experiences with raw meat, because participants had not experienced food poisoning from raw meat prepared at home or, even more likely, that they had experienced this but were not aware about the source of the gastrointestinal symptoms (Henke, Alter, Doherr, & Merle, 2020). Therefore, we believe that the availability heuristic may explain the null result of the personalised risk message: When people cannot rely on personal experiences associated with a risk, this often leads to an underestimation of the frequency of the risk, because events that are known from personal experience are also more readily available (Bier, 2017; Tversky & Kahneman, 1973). Another explanation concerns how the information is provided. Participants might have failed to correctly understand the numerical personalised risk message (Reyna & Brainerd, 2008). Although we communicated the probability information in a format recommended by the literature (i.e. frequencies, Visschers et al., 2009), it is probable that the combination of the prevalence of antimicrobial-resistant bacteria in food and frequency of personal meat consumption led to an underestimation of the transmission risk of these pathogens due to the optimism bias (Weinstein, 1989). Alternatively, it might be possible that an audio-visual video is more likely to be retained compared to a single visual text with a pictograph. Hence, providing the same risk information as a short video might lead to an increase in risk perception (Conijn et al., 2020; Garg, Camp, Connelly, & Lorenzen-Huber, 2012). Remarkably, participants evaluated the personalised risk message positively, although it did not achieve the intended effect on risk perception. This supports the assumption that the personalised risk message was processed but was not interpreted as intended. It would have been possible that the personalised risk message only yielded an effect in combination with more background information regarding antimicrobial resistance—that is, for participants who received the video on antimicrobial resistance and the personalised risk message. However, our data did not suggest this assumption. Although a combined delivery mode was successful in improving determinants of other health behaviour (Irvine, Ary, Grove, & Gilfillan-Morton, 2004; Soetens, Vandelanotte, de Vries, & Mummery, 2014), both the video and the personalised risk message did not have this effect in the context of safe food-handling to prevent the spread of antimicrobial-resistant bacteria.

According to the Risk Information Seeking and Processing Model (RISP; Dunwoody & Griffin, 2015), information seeking and processing is driven primarily by a person’s subjective assessment of the gap between someone’s own knowledge regarding a risk and what this person feels that he/she needs to know in order to respond to that risk adequately. It is possible that the video on antimicrobial resistance already closed this subjective gap and the personalised risk message did not add to this. The more positive evaluation of the information received in the control video in combination with the risk message compared to the control video without the risk message supports this assumption. There was a small difference between participants who received the educational video in combination with the risk message and participants who received the educational video without the risk message; this indicates that the personalised risk message did not provide additional information to the educational video.

Further, our results indicate that the increase in knowledge and risk perception did not lead to an increase in intention. The improvement in the determinants of intention was insufficient to achieve an increase in intention. An alternative explanation might be that our sample mainly consisted of intenders—that is, consumers who are already motivated to implement preventive food-handling measures against the spread of antimicrobial-resistant bacteria. However, the high level of intention suggests that a ceiling effect is likely to be involved—that is, the scale could only discriminate among respondents in the moderate-to-high range of intention to implement safe food-handling (Koedel & Betts, 2010). The educational video and personalised risk message may have achieved an increase in intention, but this could not be detected on our intention scale.

Our second study evaluated the effect of goal setting on the implementation of safe food-handling, coping self-efficacy and planning (action and coping planning). Our hypothesis was partially confirmed: guiding participants towards a specific goal increased the frequency of implementing preventive measures, but coping self-efficacy did not increase over time, regardless of the condition. Moreover, participants’ confidence regarding their ability to handle food hygienically even appeared to decrease after the pretest. It is likely that participants intended to implement more food-handling measures, but they may have realised that the implementation of such measures was more difficult than previously assumed. Although no specific cognitive skills are required, the measures require certain additions (e.g. soap or two cutting boards). Moreover, they may have experienced unexpected barriers like a lack of time. However, goal setting increased safe food-handling behaviour despite the decrease in coping self-efficacy. Therefore, including a BCT that successfully increases coping self-efficacy might lead to even greater behaviour change.

At posttest, participants in the experimental condition planned their food-handling behaviour more than those in the control condition. This is in accordance with the HAPA that suggests that action planning facilitates the implementation of a certain behaviour. Since coping planning also increased, it can be assumed that merely inviting participants to consider how to overcome barriers is sufficient to successfully increase coping planning. However, we must treat these findings with caution because coping planning was measured using only one item.

In both studies, participants were rather confident that they already handled food hygienically. However, after assessing various preventive measures in detail, there was a shift in a few participants’ self-evaluation from actor to intender. It is likely that participants initially overestimated their preventive behaviour when they had to assess their safe food-handling behaviour. After being aware of the six distinct measures, they were probably forced to downgrade their assessment. Interestingly, participants did not achieve progress in the HAPA phases after receiving the BCTs in both studies. Furthermore, it is important to emphasise that the HAPA phase was assessed for safe food handling in general. The phase achieved could vary depending on the specific food-handling measure the participant had in mind since intention or implementation of a behaviour differ between measures (see Appendix A, Table 1 or Appendix B, Table 1).

Another interesting point is that the majority of the sample was in the volitional phase although awareness about antimicrobial resistance in food was rather small. It might be that participants intended to implement food safety rules to avoid bacterial contamination in general, regardless of antimicrobial-resistant bacteria (e.g. because they have heard about Salmonella). The question assessing the HAPA phase for safe food-handling behaviour did not specifically refer to hygienic practices against antimicrobial resistance. Based on the theory it can be assumed that the BCTs might have yielded a greater impact if the sample had consisted only of the relevant target group (i.e. non-intenders in Study 1 vs. intenders in Study 2).

### Strengths and limitations

The main strength of the two studies in this paper was the application and testing of theory-based behaviour change techniques, which are required to ensure that effective interventions are implemented in practice. We investigated BCTs for different phases. They targeted individuals either in the motivational or the volitional phase. Moreover, the anonymous data collection promoted the accuracy and truthfulness of the data. Further, each study had its own strengths. Study 1 included a representative sample according to age and gender from the German-speaking part of Switzerland, whereas Study 2 included two different measurement points that enabled us to measure behaviour change in the same individuals over time.

A limitation of our studies is the self-reporting of safe food-handling behaviour, which might be biased owing to social desirability, although anonymous data collection should have prevented this. Even if participants reported to have executed the safe food-handling practices, this does not imply that they implemented the measures correctly. An observational study would provide useful information for this purpose. Furthermore, Study 2 was slightly underpowered which could have reduced the chance of detecting another effect besides on behaviour, coping planning and action planning. Another potential limitation involves the relatively short follow-up period in Study 2, which does not allow predictions regarding long-term adherence to behaviour change. Similarly, due to the cross-sectional design in Study 1, we do not know whether the video on antimicrobial resistance causes a change in behaviour. Moreover, the scope of both studies was constrained to consumers in Switzerland and it is likely that awareness for antimicrobial resistance differs across cultures and countries.

## Conclusion

This research demonstrated that a short educational video is a helpful tool to provide information regarding antimicrobial resistance and to increase consumers` knowledge and perceived susceptibility of risk with regard to the transmission of antimicrobial-resistant bacteria through food. Goal-setting is an appropriate BCT to improve consumers’ preventive food-handling behaviour to reduce the transmission risk of antimicrobial-resistant bacteria. The volitional process from the HAPA was influenced by action and coping planning, but not by coping self-efficacy. Hence, both video and goal setting offer the potential to target relevant determinants of safe food-handling behaviour among consumers in different motivational phases. However, there is room for future improvements in the content and its approaches in relation to positive outcome expectations in the educational video and coping self-efficacy in goal setting. Therefore, consumers might benefit from including additional BCTs, such as priming past successes or teaching specific coping strategies to overcome barriers (Michie et al., 2013). Visualisations of bacterial contamination might foster imagination and increase positive outcome expectancy by making the antibacterial effect of preventive food-handling measures visible; this may avoid people to become confused regarding the nature of the risk when threats are invisible (Slovic, Peters, Finucane, & MacGregor, 2005). Although messages regarding the prevalence of antimicrobial resistance in poultry did not increase risk perception, messages regarding the likelihood of the health risk (e.g. acute food poisoning or long-term diseases) might achieve the desired effect. Additional research is also required to examine how habits can be created to foster a long-term behaviour change, including different BCTs in a multi-component intervention.

## Supplementary Material

Supplemental Material

## Notes

1 The video was developed by the research team and is available at the following link https://doi.org/10.26041/fhnw-3409.

2 Regression analyses were additionally used to verify the results because the assumption of homogeneity of variance was violated (Powers & Xie, 2008). Linear regression analyses with the HAPA variables and information evaluation as the dependent variables and video and risk message as independent variables revealed the same significant results. Regression analyses with the knowledge subscales as dependent variables revealed significant results for general knowledge and knowledge about transmission, but not for knowledge about health risks.

3 The correlation between fish consumption and the dependent variables was calculated because fish consumption differed among the four conditions (see Table 1). There was no statistically significant correlation between fish consumption and any of the dependent variables: general knowledge (*r* = -.03, *p =* .64), knowledge about health risks (*r* = .10, *p =* .06), knowledge about transmission (*r* = .06, *p =* .26), susceptibility of risk (*r* = .06, *p =* .31), severity of risk (*r* = .11, *p =* .05), positive outcome expectancy (*r* = .06, *p =* .27), negative outcome expectancy (*r* = -.001, *p =* .98), or intention (*r* = .01, *p =* .93).

4 Age was included as a covariate because of the significant positive association between age and coping planning.

5 See Note 4.
