# Supplementary material for: Antimicrobial-resistant bacteria in food: which behaviour change strategies increase consumers’ risk awareness and preventive food-handling behaviour?
Source: Health Psychol Behav Med. 2021 Apr 20;9(1):350–79. doi: 10.1080/21642850.2021.1912609 (PMC8158281; doi:10.1080/21642850.2021.1912609)
Supplement: Supplemental Material [file RHPB_A_1912609_SM1235.docx]

**Appendix A**

**Scale validation of Study 1**

| **Table 1**  *Items and descriptive statistics of the HAPA variables in Study 1.* | | | |
| --- | --- | --- | --- |
|  | Items per variable | *M* | *SD* |
|  | *Intention* |  |  |
|  | I intend to… |  |  |
| I1 | wash the work surface and kitchen utensils (e.g. knife and cutting board) immediately after the preparation of raw meat, poultry, fish or seafood with soap. | 8.24 | 2.51 |
| I2 | use a separate cutting board and knife for raw meat, poultry, fish or seafood. | 9.21 | 1.41 |
| I3 | wash hands with soap immediately after the preparation of raw meat, poultry, fish or raw seafood. | 9.31 | 1.38 |
| I4 | check whether the meat, poultry, fish or seafood is cooked thoroughly. | 9.35 | 1.45 |
| I5 | put meat, poultry, fish or seafood that is not well cooked, back into the frying pan. | 9.38 | 1.29 |
| I6 | ensure that meat, poultry, fish and seafood are appropriately stored and chilled. | 9.53 | 0.98 |
|  | *Risk Perception - Susceptibility* |  |  |
|  | How likely do you think it is that… |  |  |
| RP1 | that food in Switzerland is contaminated with antibiotic-resistant bacteria? | 6.61 | 2.14 |
| RP2 | that you buy food which is contaminated with antibiotic-resistant bacteria? | 6.10 | 2.23 |
| RP3 | that antibiotic-resistant bacteria are transmitted to humans through food? | 7.44 | 2.07 |
| RP4 | that you prepare food which transfers antibiotic-resistant bacteria to you or your housemates? | 4.91 | 2.58 |
| RP5 | that antibiotic-resistant bacteria are spread by people who are not sick? | 6.27 | 2.50 |
| RP6 | that antibiotic-resistant bacteria are spread by you or your housemates although you do not feel sick? | 6.01 | 2.63 |
| RP7 | that consumers get sick because of antibiotic-resistant bacteria in food? | 6.98 | 2.12 |
| RP8 | that you or your housemates get sick because of antibiotic-resistant bacteria in food? | 5.67 | 2.51 |
| RP9 | that food poisoning due to antibiotic-resistant bacteria can be serious or mortal for humans? | 7.21 | 2.28 |
|  | *Risk Perception - Severity* |  |  |
|  | How dangerous do you think it is when… |  |  |
| RP10 | food is contaminated with antimicrobial-resistant bacteria? | 7.65 | 1.94 |
| RP11 | antibiotic-resistant bacteria are transferred from food to humans due to unsafe food preparation? | 7.88 | 1.80 |
| RP12 | a person is infected with antibiotic-resistant bacteria without being aware and she/he transmits these bacteria to others? | 7.36 | 2.11 |
| RP13 | people get sick because of antibiotic-resistant bacteria in food? | 7.84 | 1.80 |
|  | *Positive Outcome Expectancy* |  |  |
| PO1 | By adopting safe food-handling measures, I kill all pathogenic bacteria in food. | 6.87 | 2.32 |
| PO2 | By implementing hygiene measures, I reduce the spread of antibiotic resistance through food. | 8.50 | 1.79 |
| PO3 | By adopting safe food-handling measures, I can prevent infections. | 8.05 | 1.82 |
| PO4 | By using a separate cutting board and knife for raw meat, I can prevent pathogenic bacteria from spreading in my kitchen. | 8.17 | 2.00 |
| PO5 | By keeping meat, poultry, fish and seafood refrigerated, I reduce the reproduction of bacteria. | 8.73 | 1.57 |
|  | *Negative Outcome Expectancy* |  |  |
| NO1 | It is exaggerated with the hygiene standards in Switzerland, when I pay attention to the safe food handling. | 3.83 | 2.46 |
| NO2 | When I implement hygiene measures in the kitchen, it is too elaborative. | 2.63 | 1.88 |
| NO3 | When I adopt many safe food-handling measures, then I kill also useful bacteria. | 5.63 | 2.85 |
| NO4 | When I pay attention to hygienic handling in the kitchen, my immune system weakens because a certain amount of germs is necessary. | 3.34 | 2.48 |
| NO5 | When I wash my hands after every contact with raw meat, it takes too much time. | 2.27 | 1.93 |
|  | *Behaviour* |  |  |
| B1 | Washing the work surface and kitchen utensils (e.g. knife and cutting board) immediately after the preparation of raw meat, poultry, fish or seafood with soap. | 5.16 | 1.12 |
| B2 | Using a separate cutting board and knife for raw meat, poultry, fish or seafood. | 4.31 | 1.75 |
| B3 | Washing hands with soap immediately after the preparation of raw meat, poultry, fish or raw seafood. | 5.25 | 1.09 |
| B4 | Check whether the meat, poultry, fish or seafood is cooked thoroughly. | 5.52 | 0.86 |
| B5 | Putting meat, poultry, fish or seafood that is not well cooked, back into the frying pan. | 5.39 | 1.03 |
| B6 | Ensuring that meat, poultry, fish and seafood are appropriately stored and chilled. | 5.65 | 0.75 |
| *Note.* Behaviour items were assessed on 6-point response scales, ranging from 1 = never to 6 = always*.* Pre-intentional variables were assessed on 10-point response scales. | | | |

| **Table 2**  *HAPA items in Study 1: Results from the Mokken scale analysis (MSA, i.e. AISP, H coefficients, H^T^ coefficients, H_i_ coefficients, and Crit values), the exploratory factor analysis (EFA, i.e. factor loadings and proportion explained variance), and reliability analyses (inter-item correlations and Cronbach’s α).* | | | | | | | | | | | | | | | | | |
| --- | --- | --- | --- | --- | --- | --- | --- | --- | --- | --- | --- | --- | --- | --- | --- | --- | --- |
| Items | *AISP* | *H* | *H^T^* | *H_i_ (SE)* | Monotonicity | IIO |  | Factor | | | | | | |  | $\bar{r}$† | Cronbach’s α [95% CI] |
|  |  |  |  |  | *Crit* | *Crit* |  | 1 | 2 | 3 | 4 | 5 | 6 | 7 |  |  |  |
| *Intention* |  | .48 (.05) | .42 |  |  |  |  |  |  |  |  |  |  |  |  |  | .80 [.76-.83] |
| I1 | 2 |  |  | .41 (.05) | 0 | 0 |  |  |  |  |  | .81 |  |  |  | .47 |  |
| I2 | 2 |  |  | .53 (.05) | 0 | 0 |  |  |  |  |  | .48 |  |  |  | .69 |  |
| I3 | 2 |  |  | .57 (.05) | 0 | 0 |  |  |  |  |  | .48 |  |  |  | .73 |  |
| I4 | 2 |  |  | .50 (.07) | 0 | 0 |  |  |  |  |  |  |  | .81 |  | .63 |  |
| I5 | 2 |  |  | .50 (.06) | 0 | 0 |  |  |  |  |  |  |  | .86 |  | .61 |  |
| I6 | 2 |  |  | .42 (.06) | 0 | 0 |  |  |  |  |  |  |  | .49 |  | .51 |  |
| *Risk Perception – Susceptibility* | | |  |  |  |  |  |  |  |  |  |  |  |  |  |  |  |
|  |  | .43 (.03) | .34 |  |  |  |  |  |  |  |  |  |  |  |  |  | .86 [.84-.87] |
| RP1 | 1 |  |  | .43 (.03) | 4 | 0 |  | .66 |  |  |  |  |  |  |  | .62 |  |
| RP2 | 1 |  |  | .43 (.03) | 0 | 64 |  | .76 |  |  |  |  |  |  |  | .60 |  |
| RP3* | 1 |  |  | .43 (.03) | 0 | 107 |  |  |  |  |  |  |  |  |  |  |  |
| RP4 | 1 |  |  | .40 (.04) | 0 | 0 |  | .65 |  |  |  |  |  |  |  | .55 |  |
| RP5 | 1 |  |  | .45 (.03) | 0 | 50 |  | .76 |  |  |  |  |  |  |  | .64 |  |
| RP6 | 1 |  |  | .47 (.03) | 6 | 41 |  | .83 |  |  |  |  |  |  |  | .67 |  |
| RP7 | 1 |  |  | .43 (.04) | 0 | 0 |  | .50 |  |  |  |  |  |  |  | .62 |  |
| RP8 | 1 |  |  | .44 (.03) | 0 | 0 |  | .61 |  |  |  |  |  |  |  | .63 |  |
| RP9* | 1 |  |  | .39 (.03) | 0 | 109 |  |  |  |  |  |  |  |  |  |  |  |
| *Risk Perception – Severity* | | | |  |  |  |  |  |  |  |  |  |  |  |  |  | .89 [.87-.91] |
| RP10 | 1 |  |  | .38 (.04) | 0 | 0 |  |  |  | .81 |  |  |  |  |  | .53 |  |
| RP11 | 1 |  |  | .48 (.03) | 0 | 0 |  |  |  | .80 |  |  |  |  |  | .65 |  |
| RP12 | 1 |  |  | .41 (.03) | 0 | 0 |  |  |  | .85 |  |  |  |  |  | .59 |  |
| RP13 | 1 |  |  | .43 (.03) | 0 | 0 |  |  |  | .82 |  |  |  |  |  | .60 |  |
| *Positive Outcome Expectancy* | | |  |  |  |  |  |  |  |  |  |  |  |  |  |  |  |
|  |  | .38 (.04) | .10 |  |  |  |  |  |  |  |  |  |  |  |  |  | .70 [.64-.75] |
| PO1* | 0 |  |  |  |  |  |  |  |  |  |  |  |  |  |  |  |  |
| PO2 | 4 |  |  | .31 (.05) | 0 | 0 |  |  |  |  |  |  | .40 |  |  | .45 |  |
| PO3 | 4 |  |  | .46 (.04) | 0 | 0 |  |  |  |  |  |  | .87 |  |  | .58 |  |
| PO4 | 4 |  |  | .38 (.05) | 0 | 0 |  |  |  |  |  |  | .60 |  |  | .43 |  |
| PO5 | 4 |  |  | .39 (.05) | 0 | 0 |  |  |  |  |  |  | .63 |  |  | .45 |  |
| *Negative Outcome Expectancy* | | |  |  |  |  |  |  |  |  |  |  |  |  |  |  |  |
|  |  | .41 (.04) | .25 |  |  |  |  |  |  |  |  |  |  |  |  |  | .72 [.66-.76] |
| NO1 | 3 |  |  | .34 (.05) | 52 | 0 |  |  |  |  | .56 |  |  |  |  | .37 |  |
| NO2 | 3 |  |  | .46 (.04) | 0 | 0 |  |  |  |  | .71 |  |  |  |  | .54 |  |
| NO3* | 0 |  |  |  |  |  |  |  |  |  |  |  |  |  |  |  |  |
| NO4 | 3 |  |  | .40 (.05) | 28 | 0 |  |  |  |  | .68 |  |  |  |  | .52 |  |
| NO5 | 3 |  |  | .48 (.04) | 0 | 0 |  |  |  |  | .72 |  |  |  |  | .54 |  |
| *Behaviour* |  |  |  |  |  |  |  |  |  |  |  |  |  |  |  |  |  |
|  |  | .46 (.05) | .53 | .48 (.05) |  |  |  |  |  |  |  |  |  |  |  |  | .78 [.74-.81] |
| B1 | 2 |  |  | .36 (.05) | 0 | 0 |  |  | .66 |  |  |  |  |  |  | .63 |  |
| B2 | 2 |  |  | .53 (.05) | 0 | 0 |  |  |  |  |  | .69 |  |  |  | .68 |  |
| B3 | 2 |  |  | .50 (.06) | 0 | 60 |  |  | .71 |  |  | .30 |  |  |  | .48 |  |
| B4 | 2 |  |  | .40 (.07) | 0 | 54 |  |  | .80 |  |  |  |  |  |  | .40 |  |
| B5 | 2 |  |  | .53 (.07) | 0 | 0 |  |  | .56 |  |  |  |  |  |  | .62 |  |
| B6 | 2 |  |  | .48 (.05) | 0 | 0 |  |  | .73 |  |  |  |  |  |  | .62 |  |
|  |  |  |  |  |  |  |  |  |  |  |  |  |  |  |  |  |  |
| % explained variance | |  |  |  |  |  |  | 21.74 | 12.94 | 5.37 | 4.63 | 3.91 | 3.75 | 2.65 |  |  |  |
| *Note.* *Item excluded from the EFA and Cronbach`s alpha. Extraction method in the EFA: principal axis factoring. Rotation method: Oblique promax. Loadings below .30 are not reported here. $\bar{r}$† denotes the inter-item correlation. | | | | | | | | | | | | | | | | | |

| **Table 3**  *Knowledge items in Study 1: Response frequencies and results from the Mokken scale analysis (i.e. AISP, H coefficients, H^T^ coefficients, H_i_ coefficients, and Crit values).* | | | | | | | | |
| --- | --- | --- | --- | --- | --- | --- | --- | --- |
|  | correct | incorrect |  |  |  |  | Monotonicity | IIO |
|  | *n* (%) | *n* (%) | AISP | *H* | *H^T^* | *H_i_ (SE)* | *Crit* | *Crit* |
| *General Knowledge* |  |  |  | .39 (.04) | .58 |  |  |  |
| Antibiotic-resistant bacteria only occur in humans and animals that have been treated with antibiotics.* | 262 (79.9) | 66 (20.1) | 1 |  |  | .39 (.05) | 0 | 0 |
| Only people with a weakened immune system need to protect themselves from antibiotic-resistant bacteria.* | 289 (88.1) | 39 (11.9) | 1 |  |  | .38 (.06) | 0 | 0 |
| If the human body gets used to antibiotics, it becomes resistant.* | 75 (22.9) | 253 (77.1) | 1 |  |  | .41 (.07) | 0 | 0 |
| Only the use of antibiotics in livestock is responsible for antibiotic resistance in humans.* | 258 (78.7) | 70 (21.3) | 1 |  |  | .37 (.05) | 0 | 0 |
| Antibiotic-resistant bacteria can be transmitted from animals to humans. | 286 (87.2) | 42 (12.8) | 1 |  |  | .31 (.06) | 0 | 0 |
| People always get sick when they carry antibiotic-resistant bacteria.* | 239 (72.9) | 89 (27.1) | 1 |  |  | .42 (.05) | 0 | 0 |
| In 2018 there were fewer reports of people suffering from an antibiotic-resistant infection than in 2015.* | 125 (38.1) | 203 (61.9) | 1 |  |  | .46 (.06) | 0 | 0 |
| There is no risk of antibiotic-resistant bacteria in local foods.* | 292 (89.0) | 36 (11.0) | 1 |  |  | .40 (.06) | 0 | 0 |
| *Knowledge about Health Risks* |  |  |  | .38 (.06) | .40 |  |  |  |
| Antibiotic-resistant bacteria can cause health problems in humans and animals. | 324 (98.8) | 4 (1.2) | 2 |  |  | .56 (.15) | 0 | 0 |
| Antibiotic-resistant bacteria do not cause infections of the gastrointestinal tract.* | 225 (68.6) | 103 (31.4) | 2 |  |  | .35 (.07) | 0 | 0 |
| A person suffering from food poisoning, (e.g. by the consumption of infected raw chicken meat), can have ingested antibiotic-resistant bacteria. | 247 (75.3) | 81 (24.7) | 2 |  |  | .38 (.06) | 0 | 0 |
| Antibiotic-resistant bacteria on meat can be killed by cooking it thoroughly. | 281 (85.7) | 47 (14.3) | 2 |  |  | .40 (.07) | 0 | 0 |
| *Knowledge about Transmission* |  |  |  |  |  |  |  |  |
| Campylobacter and Salmonella are examples of antibiotic-resistant bacteria. | 150 (45.7) | 178 (54.3) | 3 | .37 (.08) | .62 | .35 (.11) | 0 | 0 |
| Antibiotic-resistant bacteria can also be transmitted through the consumption of organic food. | 275 (83.8) | 53 (16.2) | 3 |  |  | .34 (.08) | 0 | 0 |
| Safe food-handling behaviour (e.g. washing hands before food preparation) is an effective measure against the spread and the transmission of antibiotic-resistant bacteria. | 294 (89.6) | 34 (10.4) | 3 |  |  | .39 (.09) | 0 | 0 |
| A treatment with antibiotics can cause antibiotic-resistant bacteria. | 225 (68.6) | 103 (31.4) | 0 |  |  |  |  |  |
| *Note.* The response scale for the knowledge items was true, false, and I don’t know. Incorrect and ‘I do not know’ answers were merged together. *Item is incorrect. | | | | | | | | |

**Appendix B**

**Scale validation of Study 2**

| **Table 1**  *Items and descriptive statistics of the post-intentional HAPA variables in Study 2.* | | | | | | |
| --- | --- | --- | --- | --- | --- | --- |
|  |  | *Pretest* | |  | *Posttest* | |
|  | Items per variable | *M* | *SD* |  | *M* | *SD* |
|  | *Behaviour* |  |  |  |  |  |
| B1 | Ensuring that meat, poultry, fish, and seafood are properly stored and chilled (5°C). | 5.80 | 0.51 |  | 5.79 | 0.48 |
| B2 | Washing hands, cutting board, and kitchen utensils immediately after the preparation of raw meat, poultry, fish or raw seafood with soap. | 5.16 | 1.17 |  | 4.99 | 1.20 |
| B3 | Putting meat, poultry, fish or seafood that is not well cooked back into the pan and continue cooking. | 4.92 | 1.43 |  | 4.43 | 1.97 |
| B4 | Using a separate cutting board and knife for the preparation of raw meat, poultry, fish or seafood, and a separate cutting board and knife for other foods (e.g. vegetables) | 4.61 | 1.69 |  | 4.79 | 1.65 |
| B5 | Changing kitchen towels, rags and sponges once a week and washing it at 60°C. | 4.58 | 1.41 |  | 4.61 | 1.55 |
| B6 | Washing the dish sponge and dishwashing brush thoroughly with soap and hot water after cleaning kitchen utensils used to prepare raw meat, poultry, fish or seafood. | 4.54 | 1.43 |  | 4.56 | 1.45 |
| B7 | Not touching or placing the smartphone on the work surface during food preparation. | 3.69 | 1.61 |  | 4.35 | 1.60 |
|  | *Coping Self-Efficacy* |  |  |  |  |  |
|  | I believe that I can implement safe food-handling measures even if… |  |  |  |  |  |
| CS1 | I don’t see positive results. | 5.12 | 1.09 |  | 4.81 | 1.36 |
| CS2 | I am with friends and relatives who find such hygiene measures exaggerated. | 5.03 | 1.17 |  | 4.88 | 1.36 |
| CS3 | it takes time until such hygiene measures become a habit. | 4.67 | 1.23 |  | 4.51 | 1.39 |
| CS4 | even if such hygiene measures are effortful. | 4.67 | 1.29 |  | 4.57 | 1.33 |
| CS5 | I don’t have time. | 4.56 | 1.35 |  | 4.42 | 1.34 |
|  | *Action Plans* |  |  |  |  |  |
|  | I have already planned concretely… |  |  |  |  |  |
| AP1 | with which hygiene measures I can improve my food handling behaviour (e.g. of raw meat). |  |  |  | 3.81 | 1.57 |
| AP2 | how to implement safe food-handling measures. |  |  |  | 3.99 | 1.57 |
|  | *Coping Plans* |  |  |  |  |  |
| CP1 | I have already planned concretely how to deal with possible barriers when implementing safe food-handling measures. |  |  |  | 3.50 | 1.59 |
| *Note.* Behaviour items were assessed on 6-point response scales, ranging from 1 = never to 6 = always*.* Coping self-efficacy, action planning and coping planning were assessed on a 6-point response scales, ranging from 1 = don’t agree at all to 6 = fully agree. | | | | | | |

| **Table 2**  *HAPA variables at pretest in Study 2: Results from the MSA (i.e. AISP, H coefficients, H^T^ coefficients, H_i_ coefficients, and Crit values), the EFA (i.e. factor loadings and proportion explained variance), and reliability analyses (Cronbach’s α and inter-item correlations).* | | | | | | | | | | | | | | |
| --- | --- | --- | --- | --- | --- | --- | --- | --- | --- | --- | --- | --- | --- | --- |
| **Pretest** | | | | | | | | | | | | | | |
| Items | *AISP* | *H* | *H^T^* | *H_i_ (se)* | Monotonicity | IIO |  | Factor | | | |  | $\bar{r}$† | Cronbach’s α [95% CI] |
|  |  |  |  |  | *Crit* | *Crit* |  | 1 | 2 | 3 | 4 |  |  |  |
| *Behaviour* |  | .17 (.04) | .34 |  |  |  |  |  |  |  |  |  |  | .54 [.41-.65] |
| B1 | 1 |  |  | .24 (.05) | 0 | 0 |  |  | .48 |  |  |  | .22 |  |
| B2 | 1 |  |  | .25 (.05) | 0 | 200 |  |  | .56 |  |  |  | .40 |  |
| B3 | 0 |  |  | .11 (.05) | 0 | 0 |  |  | .57 |  |  |  | .18 |  |
| B4 | 0 |  |  | .16 (.07) | 0 | 0 |  |  |  |  |  |  | .45 |  |
| B5 | 2 |  |  | .24 (.05) | 0 | 119 |  |  |  | .74 |  |  | .07 |  |
| B6 | 0 |  |  | .05 (.06) | 0 | 282 |  |  |  | .30 |  |  | .43 |  |
| B7 | 2 |  |  | .15 (.06) | 0 | 0 |  |  |  |  | .88 |  | .23 |  |
| *Coping Self-Efficacy* | | 53 (.06) | .15 |  |  |  |  |  |  |  |  |  |  | .83 [.78-.87] |
| CS1 | 1 |  |  | .58 (.06) | 0 | 0 |  | .73 |  |  |  |  | .70 |  |
| CS2 | 1 |  |  | .38 (.10) | 0 | 0 |  | .37 |  |  |  |  | .43 |  |
| CS3 | 1 |  |  | .58 (.06) | 0 | 0 |  | .85 |  |  |  |  | .70 |  |
| CS4 | 1 |  |  | .57 (.07) | 0 | 0 |  | .84 |  |  |  |  | .70 |  |
| CS5 | 1 |  |  | .54 (.06) | 0 | 0 |  | .69 |  |  |  |  | .65 |  |
| % explained variance |  |  |  |  |  |  |  | 28.71 | 8.206 | 5.15 | 4.36 |  |  |  |
| *Note.* Extraction method for EFA: principal axis factoring. Rotation method: Oblique promax. Loadings below .30 are not reported. $\bar{r}$† denotes the inter-item correlation. | | | | | | | | | | | | | | |

| **Table 3**  *HAPA variables at posttest in Study 2: Results from the MSA (i.e. MSA, i.e. AISP, H coefficients, H^T^ coefficients, H_i_ coefficients, and Crit values), the EFA (i.e. factor loadings and proportion explained variance), and reliability analyses (inter-item correlations and Cronbach’s α).* | | | | | | | | | | | | | | |
| --- | --- | --- | --- | --- | --- | --- | --- | --- | --- | --- | --- | --- | --- | --- |
| **Posttest** | | | | | | | | | | | | | | |
| Items | *AISP* | *H* | *H^T^* | *H_i_ (se)* | Monotonicity | IIO |  | Factor | | | |  | $\bar{r}$† | Cronbach’s α [95% CI] |
|  |  |  |  |  | *Crit* | *Crit* |  | 1 | 2 | 3 | 4 |  |  |  |
| *Behaviour* |  | .22 (.04) | .24 |  |  |  |  |  |  |  |  |  |  | .62 [.51-.71] |
| B1 | 1 |  |  | .34 (.06) | 0 | 88 |  |  |  | .71 |  |  | .32 |  |
| B2 | 1 |  |  | .30 (.06) | 0 | 117 |  |  |  | .53 |  |  | .55 |  |
| B3 | 0 |  |  | .13 (.07) | 0 | 627 |  |  | .56 |  |  |  | .10 |  |
| B4 | 1 |  |  | .30 (.06) | 0 | 0 |  |  |  |  | .31 |  | .49 |  |
| B5 | 1 |  |  | .37 (.05) | 0 | 369 |  |  |  | .47 | .35 |  | .26 |  |
| B6 | 0 |  |  | .21 (.06) | 41 | 189 |  |  |  |  | .55 |  | .52 |  |
| B7 | 1 |  |  | .30 (.05) | 0 | 169 |  |  |  |  |  |  | .31 |  |
| *Coping Self-Efficacy* | 1 | 58 (.06) | .08 |  |  |  |  |  |  |  |  |  |  | .86 [.81-.89] |
| CS1 | 1 |  |  | .60 (.07) | 0 | 0 |  | .90 |  |  |  |  | .70 |  |
| CS2 | 1 |  |  | .53 (.08) | 0 | 0 |  | .75 |  |  |  |  | .61 |  |
| CS3 | 1 |  |  | .55 (.07) | 0 | 0 |  | .53 |  |  |  |  | .64 |  |
| CS4 | 1 |  |  | .64 (.06) | 0 | 0 |  | .77 |  |  |  |  | .77 |  |
| CS5 | 1 |  |  | .56 (.08) | 0 | 0 |  | .31 |  |  | .68 |  | .65 |  |
| *Planning* | | 82 (.03) | .22 |  |  |  |  |  |  |  |  |  |  | .93 [.90-95] |
| AP1 | 1 |  |  | .82 (.03) | 0 | 0 |  |  | .94 |  |  |  |  |  |
| AP2 | 1 |  |  | .86 (.02) | 0 | 0 |  |  | .84 |  |  |  |  |  |
| CP1 | 1 |  |  | .79 (.04) | 0 | 0 |  |  | .62 |  | .45 |  |  |  |
| % explained variance |  |  |  |  |  |  |  | 36.79 | 8.21 | 5.04 | 3.32 |  |  |  |
| *Note.* Extraction method for EFA: principal axis factoring. Rotation method: Oblique promax. Loadings below .30 are not reported. $\bar{r}$† denotes the inter-item correlation. | | | | | | | | | | | | | | |

**Appendix C**

**Personalized risk message in Study 1**

Samples taken in Swiss slaughterhouses have shown that up to 60% of poultry meat is contaminated with antibiotic-resistant Campylobacter jejuni bacteria. This implies that 6 out of 10 chicken legs carry antibiotic-resistant bacteria (see figure below).


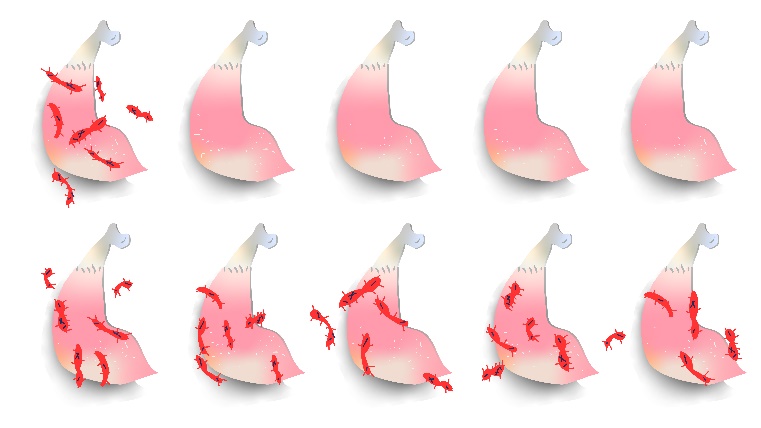


We use poultry as an example, but other meat and food can also be contaminated with antibiotic-resistant bacteria. Poultry meat is very popular in Switzerland. A Swiss person buys on average 7.4 kg poultry per year. This accounts for a quarter of the whole meat consumption and corresponds to about 49 chicken breasts. You reported to prepare poultry *[e.g.* once a week*]*. Prepare and store foods hygienically to eat it without concern.

**Appendix D**

**Definition of antimicrobial resistant bacteria in Study 2**

Antibiotic resistance implies that antibiotics no longer defeat bacteria. Antibiotic treatments do not kill antibiotic-resistant bacteria and the course of the disease is prolonged. Like non-resistant bacteria, antibiotic-resistant bacteria can be transmitted between humans and animals via different pathways. Antibiotics are also used in animal husbandry. Therefore, antibiotic-resistant bacteria can spread in the kitchen via food products (e.g. raw meat, raw poultry, raw fish or raw seafood) and can then be transmitted to humans. Food produced in Switzerland is also contaminated with pathogenic bacteria (e.g. Campylobacter is present in 40% of poultry meat), which can be resistant against antibiotics. Antibiotic-resistant bacteria may infect people and impede the treatment of the infections they cause. Hygienic food-handling measures must be implemented in the kitchen to ensure that food can be consumed safely. The Federal Food Safety and Veterinary Office recommends appropriate separation, washing, cooking, and chilling when preparing food to prevent the spread of pathogenic bacteria.

**Appendix E**

**Advices including an illustration and a short text in Study 2**

Following illustrations were provided in the experimental condition. Corresponding to the goals they set, each respondent received two illustrations with explanations.

| 1)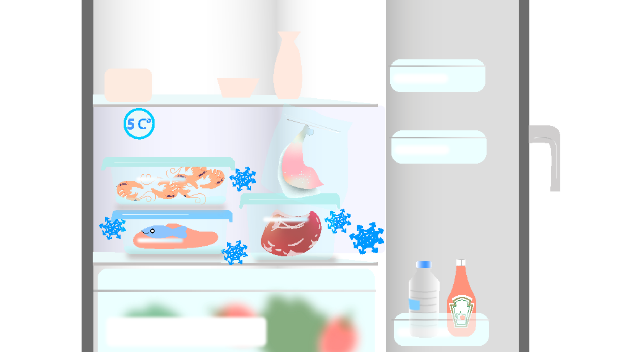 |
| --- |
| In order to achieve your goal successfully, we make the following recommendations: Store raw meat, raw poultry, raw fish, and raw seafood in a sealed container on the bottom shelf of the fridge. The bottom shelf above the vegetable compartment is usually the coldest place in the refrigerator and therefore ideal to store meat. |
| 2)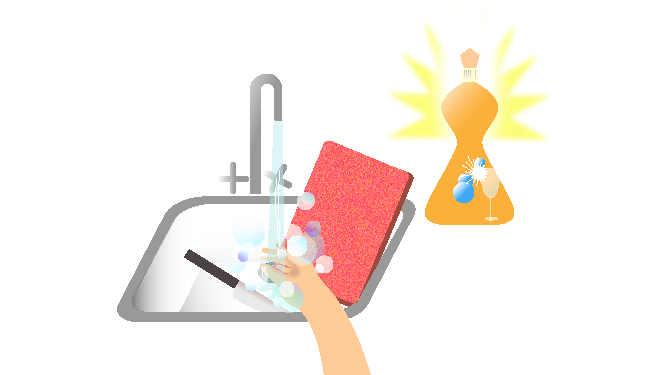 |
| Place the dishwashing detergent next to the sink. Wash used cutting board and kitchen utensils also with soap and warm water before putting them in the dishwasher. The resistant germs can spread in the dishwasher otherwise. Wash your hands before touching anything in the kitchen. |
| 3)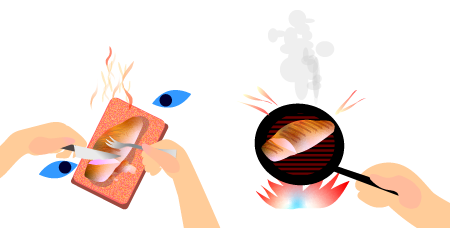 |
| Pierce into the thickest part of the meat to check whether the meat has been cooked thoroughly. The juices should run clear. Experts recommend a meat thermometer to measure the internal temperature of meat because checking colour of the meat is no indication that all germs have been killed. Use separate kitchen utensils, e.g. a fork, to pierce the meat. Wash the kitchen utensils thoroughly with soap and warm water immediately after using them. |
| 4)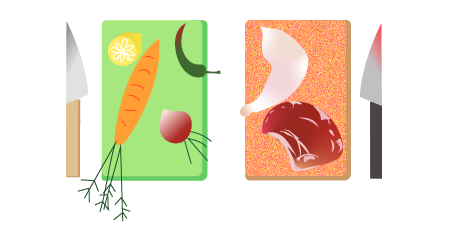 |
| Use cutting boards of different colours: one cutting board for vegetables and fruit and another cutting board for the raw animal products (meat, poultry, fish, or seafood) you are preparing. Inform your family members or housemates which colour is used for which food. |
| 5)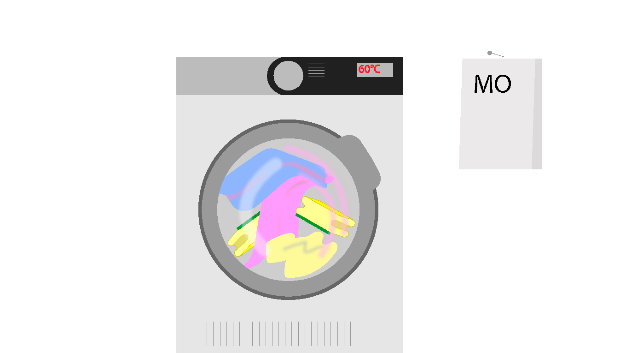 |
| Select one day of the week on which you will change used kitchen towels and sponges. Wash these at a temperature of 60°C. A hot wash will kill possible antibiotic-resistant bacteria. |
| 6)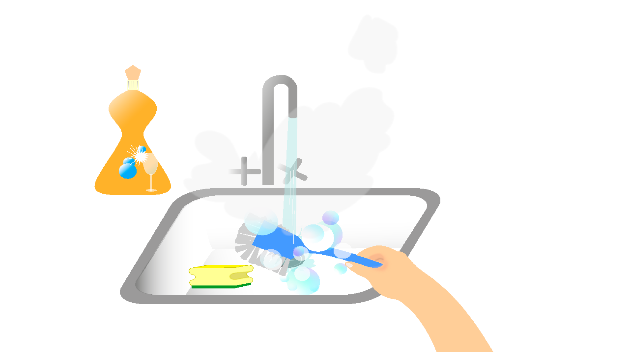 |
| Dish sponges and brushes are a bacteria hotbed. Antibiotic-resistant bacteria can also land on dish sponges and dishwashing brushes and then be further spread in your kitchen. After washing kitchen utensils used to prepare raw meat, poultry or fish, rinse sponges and brushes thoroughly with hot water (at least for 20 seconds). Let the sponges dry completely afterwards. Place the dishwashing detergent next to the sink to prevent touching anything before washing up. Dishwasher-safe washing brushes can be washed in the dishwasher’s cutlery basket. |
| 7)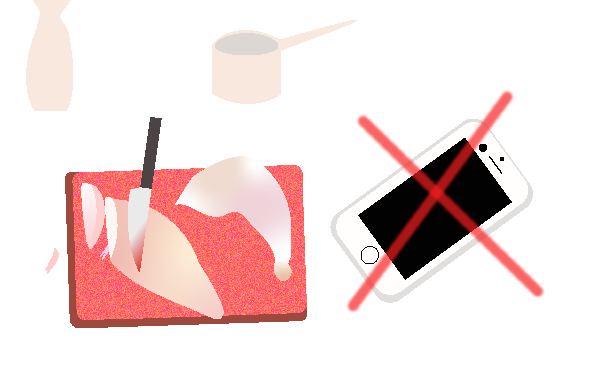 |

Smartphones are crawling with bacteria. In order to prevent antibiotic-resistant bacteria on your display, it is important to place the smartphone outside the kitchen during the preparation of raw poultry, raw meat, raw fish, or raw seafood. Wash your hands thoroughly with soap and warm water before touching your smartphone after preparing food.
